# Beyond borders: Does firm-level exposure to state and local paid sick leave mandates lead to intra-firm spillovers?

**DOI:** 10.1002/pam.70061

**Published:** 2025-10-23

**Authors:** Daniel Schneider, Kristen Harknett

**Affiliations:** 1 Harvard Kennedy School, Cambridge, Massachusetts, USA; 2 Department of Social and Behavioral Sciences, University of California, San Francisco, California, USA

## Abstract

States and localities have passed labor standards to raise the floor on job quality, including mandating employer provision of paid sick leave (PSL). However, contemporary political polarization makes the enactment of national labor standards and of state standards in many states unlikely. Prior literature has examined how labor standards may “spill over” spatially to affect firms, jobs, and workers in nearby places who are not covered by labor standards. We motivate and empirically examine the potential for “intra-firm spillovers.” We argue that multi-state firms facing a patchwork of labor standards may align company labor practices with the most stringent regulatory environments that they face given the geographic distribution of their establishments. To test this possibility, we take advantage of new employer–employee linked data from The Shift Project and focus on the case of paid sick leave. We find that state and local paid sick leave mandates spillover through multi-state employers to provide workers in places without mandates effective access to paid sick leave, and these findings survive a placebo test using other fringe benefits. Companies act as conduits through which the reach of local mandates that raise the floor on job quality are expanded to a broader set of workers.

## INTRODUCTION

Work has become more precarious in the United States over the past 50 years (Hacker, 2006; Kalleberg, 2011), with jobs increasingly characterized by low and stagnant wages (Ross & Bateman, 2019), unstable and unpredictable work schedules (Lambert, 2008; Schneider & Harknett, 2019), and a lack of basic benefits such as paid family and medical leave and paid sick leave (Heymann et al., 2009; Lovell, 2004). In the face of largely stagnant federal labor standards (Alexander & Haley-Lock, 2015; Baker, 2020), state and local policymakers have sought to raise the floor by enacting labor standards governing minimum wages, fair scheduling, and paid sick leave (Galvin, 2019). A large body of research examines the effects of such labor standards on the workers who are covered by these laws (i.e., Callison & Pesko, 2022; Cengiz et al., 2019; Harknett et al., 2021). However, these laws leave millions of workers uncovered and, given an increasingly polarized polity (de Bruin et al., 2020; Pew Research Center, 2014), with few prospects for access to higher standards via either local, state, or federal action.

However, the incomplete patchwork of local and state labor standards may shape the working conditions of workers in less regulated environments through labor policy spillovers. An important line of research examines the spatial spillover effects of minimum wage laws. Because jurisdictional boundaries do not delimit geographically integrated labor or product markets, it is possible that both worker (McKinnish, 2017) and business (Dharmasanker & Yoo, 2023) mobility across these boundaries in response to minimum wages would place upward pressure on wages in non-covered places (Jardim et al., 2024).

In addition to these spatial spillovers, researchers have conjectured that local labor standards mandates might lead multi-state firms to design common benefits packages that comply with state/local standards but are applied to all workers at the firm (Clemens, 2021), which is consistent with company-wide wage setting (Derenoncourt & Weil, 2025; Hazell et al., 2022). Given employer concentration and the prevalence of multi-state firms (Berger et al., 2019; Stansbury, 2021), many large firms encounter a variety of state- and local-level regulatory environments. Faced with this patchwork, firms may align company human resource management practices with the most stringent regulatory environments that they face given the distribution of their establishments across the United States. We term this possibility *intra-firm spillover*. While voluntary extension of benefits beyond the location required incurs extra labor costs, firms may still adopt such an approach because it could simplify HR systems, because firms learn that there is a business-case for voluntary provision via their exposure to mandated provision, and/or to reduce inequities within the organization. Therefore, firms may act as conduits for spillover. Alternatively, firms may engage in what we term *narrow compliance* in which they narrowly align company human resource management practices with prevailing local regulations in an effort to minimize direct payroll costs (Lambert, 2008). However, to our knowledge, prior research has not directly tested for such intra-firm spillovers of sub-national labor policy.

We do so by focusing on the case of state and local paid sick leave mandates. While paid sick leave appears to have beneficial effects on workers (Callison & Pesko, 2022; Callison et al., 2023; Schneider, 2020) and public health (Pichler et al., 2020, 2021), the United States lacks a federal mandate. However, workers in many states and localities are covered by paid sick leave mandates (A Better Balance, 2024; National Partnership for Women & Families, n.d.). We further focus on the service sector as a strategic site for this work. Service sector work in the U.S. is well known for its poor working conditions, and service sector workers are more likely to lack access to important job-related resources like PSL (Gould & Schieder, 2017; Schneider & Harknett, 2020a). Because these jobs are customer facing and in heavily trafficked workplaces, a lack of PSL has widespread implications for public health (Pichler & Ziebarth, 2017).

To adjudicate between a pattern of narrow compliance and that of intra-firm spillover, we focus on workers who are *not covered* by state or local paid sick leave mandates and who are employed by large, multi-state employers. We ask if these workers are more likely to have access to PSL when they work for firms with greater exposure to PSL mandates across their national business operations by dint of the geographic distribution of their establishments and workers.

We use a set of linked-employer-employee data collected from workers at large retail and food service firms in the United States to take up this question. We merge time-place varying data on PSL mandates with administrative data on the geographic location of every establishment/store and headcount for a set of 135 large multi-state service sector firms to construct time-varying measures of firm-level exposure to PSL mandates across their business operations and then merge these firm-month level data to surveys of 31,804 hourly service sector workers collected by the Shift Project using online recruitment between 2018 and 2021 who were employed in jurisdictions without PSL mandates in effect and by these same 135 firms.

We find consistent evidence of intra-firm spillovers. Workers employed in non-PSL mandate places are significantly more likely to report having access to PSL at work when employed at firms that have greater exposure to PSL mandates in their operations. We then subject this finding to a series of logical or falsification tests. We show that, as expected, firm-level exposure to PSL mandates does not shape worker access to other benefits. Further, these effects are most pronounced for workers where paid sick leave mandates are likely to be most salient for executives and upper management (Zhang, 2022), captured by firms that are headquartered in cities or states that mandate paid sick leave. Additionally, these effects are much weaker for firms that lack centralized control of labor practices, captured by franchising. In sum, we provide new evidence that sub-national labor standards may have intra-firm spillovers, shaping workers’ access to important benefits, such as PSL, beyond the borders of enacting jurisdictions. In all, we articulate the idea of intra-firm spillovers and provide some of the first empirical evidence of these processes, significantly adding to our understanding of labor policy spillovers.

## SPILLOVER EFFECTS FROM LABOR REGULATIONS

Sub-national regulations may have spillover effects, as local and state laws shape the behavior of regulated firms that are not covered by the law. The legal scholar Heather Gerken has observed that disputes regarding policy boundaries between the states and the federal government are common and well understood as predictable problems of federalism, but that when one state’s action affects firms in another state, this creates a new kind of problem, one of horizontal federalism (Gerken & Holtzblatt, 2014).

In the domain of labor policy, prior research has taken up the case of such spatial spillovers and focuses on the minimum wage. Research in this area has found that minimum wage laws effectively raise wages for covered workers (e.g., Cengiz et al., 2019; Dube & Lindner, 2024). Such mandated wage effects could then also result in spatial spillovers by changing the dynamics of employer demand for and worker supply of labor. Workers could migrate/commute across jurisdictional boundaries to jobs covered by higher wages, leaving a dearth of supply in adjacent areas and/or firms could locate establishments outside of covered areas in response to minimum wages leading to greater labor demand (Jardim et al., 2024). There is recent evidence consistent with these processes. Workers appear to increase commuting in response to minimum wage standards (McKinnish, 2017), firms are more likely to site new establishments outside of covered geographies and less likely within covered geographies (Dharmasankar & Yoo, 2023), and, for large minimum wage increases, wages rise and hours decline outside of covered areas, consistent with spatial spillovers (Jardim et al., 2024).

## INTRA-FIRM SPILLOVER

In addition to this process of spatial spillover, prior literature has conjectured that firms exposed to local and state labor standards in parts of their business operations might be induced to adopt mandated wage levels or benefits across their workforces (Clemens, 2021). There is anecdotal evidence for the expectation of spillover from both policy advocates and corporate leaders. For instance, in the area of secure scheduling ordinances, one advocate described a policy strategy of focusing on relatively small though progressive cities as being based in part on the idea that

if a company has to set the system for one policy, it’s in their interest to just lift the floor for everybody… Once you show you can do these things, they’re [firms] going to have a problem if they don’t give it to everybody else. But also, it’s easier within their system to set it across the board.

(personal communication, December 7, 2022)

A number of mechanisms could account for an effect of firm exposure to local labor standards on firm offerings to non-covered workers. We describe how such intra-firm spillovers could be produced by three mechanisms.

First, firms may exhibit intra-firm spillovers and extend benefits in locations where not legally required to do so because of the value of simplified and streamlined human resource (HR) practices and policy and compliance tracking. HR and accounting systems are often shared across units in a firm (Endenich et al., 2011), and these common systems may then create some impetus for uniformity in employment practices. Firms operating in a multitude of policy environments, some with sub-national labor standards in place and some without, may elect to comply with the most stringent of environments. Recent comments by the director of retail operations at the firm UNTUCKit made this logic explicit, as he explained his view that best practice is to accept whatever is the most restrictive policy as general practice, with the benefit that this streamlines complexity by ensuring that stores adhere to a baseline of compliance (Paid Sick Leave Laws, 2022). Indeed, this logic is made explicit in a 2019 piece in the Society for Human Resource Management’s *HR Magazine*, which dispensed advice on “how to comply with new state and local laws,” highlighting that, “Companies must make a choice: Either pay everyone at the highest wage rate to ensure compliance with all laws, or set pay on a jurisdiction-by-jurisdiction basis” (Deschenaux, 2019).

Second, firms may also be motivated to extend mandated labor regulations more broadly than required if they observe positive effects for their business from a particular regulation. Even though the retail industry is known for poor working conditions, some firms engage in “high-road” practices (Appelbaum et al., 2003; Carré & Tilly, 2017), voluntarily offering employees a range of fringe benefits, including paid sick leave. In part, these high-road practices emerge from the need to align business practices with local, state, and federal mandates such as minimum wage requirements, local fair workweek regulations, or local paid sick leave mandates. However, there is some evidence that high-road practices confer benefits for business in terms of improved operation execution, reduced employee turnover, and even higher sales and profits (Ton, 2014). In one recent and rigorous study, an experimental intervention in the Gap clothing stores that increased schedule stability led to improvements in employee turnover and sales rates (Williams et al., 2018). In a separate example, when San Francisco passed the first domestic partner benefit regulation, they articulated a business case for offering these benefits; namely, that companies that offer such benefits could expect to attract the most qualified workers (Reich et al., 2014). Subsequently, competitive pressures within sectors fueled a race to the top in which companies voluntarily extended benefits (Raeburn, 2004). In the same vein, firms located in states with mandatory secure scheduling policies, paid leave, or higher minimum wages may be motivated to extend this high-road practice to establishments located in states without mandates if they expect benefits in terms of employee retention or enhanced productivity.

Finally, firms may come under pressure for horizontal equity if workers in one establishment have a particular benefit and nearby workers do not. Research on companies’ voluntary adoption of domestic partner benefits cites evidence that horizontal equity concerns were one driver of this process (Raeburn, 2004). A long-standing literature in sociology has examined how individuals make relative comparisons to various reference groups (Jencks et al., 1972; Merton & Rossi, 1957; Veblen, 1899). One important mechanism that produces these effects is the experience of relative deprivation (Stouffer et al., 1949). In the workplace, providing information to workers that reveals them to be earning low wages relative to coworkers reduced satisfaction (Card et al., 2012). Workers who experience more unstable and unpredictable scheduling practices than their coworkers also report much lower job satisfaction and much higher turnover intentions, net of their actual level of exposure to schedule instability (Storer, 2022). That relative deprivation in job quality reduces job satisfaction and increases turnover intentions may then provide an impetus to firms to ensure a degree of horizontal equity in labor practices.

All three of these—the benefits of standardizing HR offerings, learning about the benefits of high-road practices, and ensuring horizontal equity—are potential causal mechanisms that could produce intra-firm spillovers. These mechanisms could lead firms with significant exposure to local mandates to offer the required job amenities more broadly, consistent with a process of intra-firm spillover, in which better job quality access spills over within-firms to workers not covered by mandates.

## NARROW COMPLIANCE: DOING NOTHING EXTRA

However, there is also good reason to expect that, rather than engaging in activities that lead to this kind of intra-firm spillover, multi-state firms operating both in areas that do and do not mandate higher labor standards—be they minimum wage, paid sick leave, stable schedules, or other aspects of job quality—may engage in a strategy of narrow compliance, providing the mandated job benefit only to those workers to whom mandates apply. A strategy of narrow compliance is in keeping with evidence that a wide range of firm practices are oriented toward the goal of minimizing labor costs (e.g., Lambert, 2008).

This institutional logic of labor-cost-minimization can be traced across hourly wages, work scheduling practices, and the provision of fringe benefits. Financialization and the accompanying shareholder pressure to maximize profits has exerted downward pressure on wages and contributed to wage stagnation (Palladino, 2021). An emphasis on minimizing labor costs is also a driving force behind just-in-time scheduling practices, prevalent in the service sector, which are designed to closely align staffing with customer demand (Lambert, 2008). Here, companies will often cancel shifts or cut them short to avoid any surplus staffing and the accompanying payroll costs. Firms have also followed a narrow economic and cost-minimizing logic in reducing full-time employment in favor of part-time to avoid costs associated with Affordable Care Act health-coverage mandates (Kattih et al., 2021; Mulligan, 2020). Further, although workers in 12 states and Washington, DC, as well as in 22 localities were covered by paid sick leave mandates (A Better Balance, 2024; National Partnership for Women & Families, n.d.), nearly half of workers at large national service sector firms do not have sick leave benefits, suggesting that many employers forego offering this benefit unless it is required (Schneider & Harknett, 2020a).

Further evidence supporting the expectation of narrow compliance comes from the literature on widespread noncompliance with labor laws even in the locations and for the workers they are designed to cover (Bernhardt et al., 2013). Some studies have estimated that between 17% and 25% of low-wage workers are paid less than the legally mandated minimum wage (Galvin, 2016; Wage and Hour Division, 2014). Further, compliance with secure scheduling and PSL mandates appears limited (Lambert & Haley, 2021; Petrucci et al., 2021; Tsoukalas et al., 2021). Further evidence that firm decisions are influenced by narrow economic cost-benefit ratios comes from research that firms are much more likely to violate labor laws when costs of compliance are high and costs of noncompliance are low (Clemens & Strain, 2020; Galvin, 2016).

The expectation of narrow compliance is buttressed by the active product market for tailored compliance tools. For instance, Deputy, a work scheduling software company, developed a set of tools tailored specifically to each Secure Scheduling jurisdiction, which each have slightly different rules and regulations, aiming to technologically facilitate a strategy of narrow compliance (Deputy, 2022).

This work suggests that, at best, firms may follow a kind of narrow compliance in which they closely match firm policies to prevailing regulations with the consequence of having heterogeneous labor practices across place. In sum, companies can be expected to engage in behaviors that minimize labor costs. This leads to a hypothesis of narrow compliance.

## SALIENCE AND CONTROL

The causal mechanisms outlined above that may give rise to intra-firm spillover (simplification, learned-business-case, and horizontal equity) may all operate more strongly when the salience of state and local mandates is higher. We argue that one such source of heightened salience is the location of firm headquarters in a city or state that has passed and implemented a local labor standard. Hypothetically, when corporate headquarters are located in states with paid sick leave legislation, corporate leaders may have greater familiarity with these laws and a greater acceptance of their rationale and provisions. If corporate leaders are headquartered in locations where these laws are in place, this may increase the perceived legitimacy of such laws and foster a belief that offering this fringe benefit is or could be a standard business practice.

Innovative recent research by Zhang (2022) found that firms headquartered in states that passed bans on the use of affirmative action by universities and in state government hiring had significantly slower growth in their hiring of Black managers following passage, suggesting both a spillover effect from regulations governing public sector action to the private sector and the role of headquarters location in moderating this spillover. In the case at hand, we would expect that intra-firm spillover would be more pronounced when firms were headquartered in cities or states with PSL mandates.

In contrast, these causal mechanisms of intra-firm spillover should all operate much more weakly in firms that exercise limited corporate control over local labor practices. In the service sector, franchising is an important source of such variation in corporate control. Franchisors (the parent company) closely regulate many aspects of franchisee (the local operator) behavior, including branding, store design, and products (Elmore & Griffith, 2021). However, in part to evade the legal standard of joint employer liability, in which the franchisor would be held jointly liable for illegal activity by the franchisee, franchise agreements rarely dictate labor practices in terms of wages, schedules, or paid leave (Ellmore & Griffith, 2021). While a small body of research has assessed the consequences of this form of employment fissuring (Weil, 2014) for job quality (Lakhani, 2022) and compliance with labor standards (Ji & Weil, 2015), it also follows that this weaker control exercised by the parent company (franchisor) over individual establishments should attenuate the mechanisms of intra-firm spillover. Essentially, rather than a single entity being exposed to varying degrees of state and local mandates, and then acting to simplify HR systems, implement a learned business case for higher-road approaches, or ensure horizontal equity across workers, franchising results in many small operators who exercise control over labor practices but are not exposed to the same patchwork of standards given their small and localized operations. In sum, we would expect to see less evidence of intra-firm spillover for firms that are wholly or partially franchised.

## THE CASE OF PAID SICK LEAVE

The United States is unique among developed countries in lacking a federal guarantee to paid sick leave, leaving many workers without the ability to take time off from work when sick without losing pay or even jeopardizing their job (Heymann et al., 2021). Workers in low-wage occupations like retail and food service are disproportionately represented among those lacking access to paid sick leave benefits (MacLean et al., 2020). For workers in the service sector, lacking access to paid sick leave creates pressure to work while sick (DeRigne et al., 2016), and, given that these workers are often employed in high density, indoor workplaces, therefore poses a threat to public health (Lovell, 2004).

In the absence of a federal paid sick leave guarantee, several U.S. states and localities have passed paid sick leave mandates at a more local level. While the specific provisions of these state and local laws vary, in general paid sick leave mandates require private sector employers with at least 25 workers to provide paid sick leave to their employees, with a general rate of accrual of approximately 1 hour of paid leave for every 40 hours worked, the ability to roll over up to 40 hours of accrued paid leave from year to year, and with eligibility for accrual beginning at employment and allowance for the use of accrued leave after 90 days on the job. State and local paid sick leave mandates in the United States have an employer liability model in which employers bear the modest costs of these requirements.

Prior research has documented the direct effects of PSL mandates on covered workers, finding increases in reported access to PSL and reductions in working while sick (Callison & Pesko, 2022; Colla et al., 2014; Maclean et al., 2020; Romich, 2017; Schneider, 2020), positive effects on worker health and preventative care (Callison et al., 2023; Slopen, 2023), and improvements in economic security (Slopen, 2024). Expansions in PSL also appear to have beneficial effects on public health (Pichler et al., 2020, 2021).

San Francisco passed the first PSL mandate in 2006 (National Partnership for Women & Families, 2023). As of May 2021 (the end of our analysis period), PSL had been enacted and implemented by 12 states and Washington, DC, as well as by 22 localities (A Better Balance, 2024; National Partnership for Women & Families, n.d.), with one additional state, NM, having passed but not implemented PSL laws by May of 2021 (National Partnership for Women & Families, n.d.). This includes several Texas counties that passed and implemented paid sick leave laws that were in effect during our observation period. However, shortly afterwards, in April of 2021, these laws were pre-empted by a state-level law (A Better Balance, 2024). In all, the share of workers covered by a state PSL law rose from 1% in 2014 to 15% in 2015 to 28% in 2019 (Pomeranz et al., 2022). This expansion of PSL mandates creates significant heterogeneity over time and between states in the period 2017 to 2021. Figure 1 shows the geography of PSL mandate coverage as of 2021.

## DATA AND METHODS

We marshal several linked data to construct the necessary data: (1) individual-level survey data collected by the Shift Project from hourly workers in the United States employed at 135 of the largest firms in the service sector, (2) The National Partnership for Women & Families’ reports of state and local paid sick leave mandates, (3) The ReferenceUSA database of store/establishment locations and headcounts, (4) Franchise Disclosure Documents that detail the number of franchised and company-owned establishments by firm, and (5) publicly-available address information on the geographic location of the corporate headquarters of each of the 135 firms in the data. We merge firm-level measures of exposure to PSL mandates, of franchising, and of head-quarters location to the individual-level survey data from the Shift Project at the firm-month level.

### Shift Project data

Our key question requires worker-level micro-data that meet three requirements: (a) direct worker reports of access to paid sick leave at their job; (b) geographic identifiers that situate workers within county and state; and (c) employer identifiers that allow the respondent to be linked to aggregate level data on their employer’s exposure to PSL mandates across the firm’s national business operations, franchise structure, and corporate headquarters location. This is a demanding set of requirements for any data set. In the United States, few large-scale data sets collect worker reports of access to paid sick leave. Still less common are geographic identifiers that identify county and state of work outside of the largest counties in the United States. Finally, almost no data in the United States provides an employer–employee link. The most well-known exception, the LEHD, provides only partial national coverage and lacks measurement of access to PSL.

To satisfy these data requirements, we turn to the Shift Project survey data. The Shift Project collects data from hourly workers employed at large firms in the service sector in the United States. The data have been collected in biannual repeated cross-sections since 2017. These data contain worker reports of access to paid sick leave, geographic identifiers, and employer identifiers that link each respondent to one of 135 large firms in the service sector.

The Shift Project data are collected using a novel sampling and recruitment approach. The Shift Project uses Meta as both sampling frame and recruitment device. Using Meta’s sophisticated adtargeting platform, the Shift Project identifies Facebook and Instagram users employed at each of 135 of the largest firms in the service sector in the United States. This approach allows for the creation of data clustered by identifiable employer, a sampling frame which is otherwise unavailable in the United States. The Shift Project then recruits these respondents to complete an online survey using targeted paid advertisements, with separate advertisements delivered to company-specific audiences of potential respondents. Facebook/Instagram users see this as sponsored content in their newsfeeds. Those who click on the content are redirected off Facebook/Instagram to an online Qualtrics survey. Respondents are asked to consent to participation and then to complete the survey. These methods are described in detail by Schneider and Harknett (2022), who also described a set of tests for bias.

We draw on data collected in Waves 3 through 10 of the Shift Project survey, covering the period from September 2017 through May of 2021.^1^ We limit the sample to respondents who completed the survey and use the Amelia package in R to impute missing data due to item non-response, creating 10 imputations. We construct weights to correct for differential response to the survey on observable demographic characteristics. To construct sample weights, we use data on service-sector workers surveyed in the American Community Survey. We stratified samples by intersections of age, race/ethnicity, gender, and industry and construct weights so that the proportion in each intersection in our sample resembled that in the benchmark data.

### State and local paid sick leave mandates

To construct measures of firm-level exposure to PSL mandates, we create state-by-month and county-by-month measures of paid sick leave mandates that apply to hourly service-sector workers in the U.S. employed at large firms. Data on the effective dates of these mandates is drawn from the regular annual reports of state, city, and county paid sick leave laws released by the National Partnership for Women & Families (n.d.).^2^

### Store/establishment locations and headcounts

To gauge firm-level exposure to PSL mandates nationally, we draw on proprietary data constructed by ReferenceUSA that contains establishment-level data for firms in the United States. For each establishment, the ReferenceUSA dataset contains the address of the establishment. In addition, for each establishment, the data contains the number of workers employed at the job site. In all, we draw on ReferenceUSA data for 378,003 establishments/stores of the 135 large service sector firms in which the Shift Project respondents are employed.

### Key variables

We use these data to construct three key variables—workers’ reported access to paid sick leave, workers’ coverage by a PSL mandate, and the extent of each firms’ exposure to PSL mandates. We make additional use of two moderating variables—firm headquarter location and firm franchising organization. We describe our controls in the Appendix.^3^

#### Worker access to paid sick leave

Our dependent variable is derived from the Shift Project data. Workers are asked: “Please look at the following list of benefits that employers sometimes make available to their employees. Which of the benefits on this list can you receive as part of your job at [EMPLOYER NAME]? Please mark all that apply.” The response options contain a standard list of employee benefits, with the first response option being “paid sick days.” We code workers as “1” if they report being able to receive “paid sick days” and as “0” otherwise.^4^

#### Presence of PSL mandate

We use the linked PSL mandates file and the Shift Project survey data file to define variables that categorize respondents in terms of employment in a place at a time with a PSL mandate in effect. We use these to define sample inclusion rules, focusing only on respondents who are *not covered* by mandates. We describe the creation of this indicator in more detail in the Appendix.

#### Firm exposure to PSL mandates

We define two key independent variables from the conjunction of the PSL mandates database and the ReferenceUSA database. First, we calculate the share of each firm’s *establishments* in each month that are in states or counties with PSL mandates in effect. Second, we calculate the share of each firm’s *workers* in each month who are in states or counties with PSL mandates in effect. We use these two alternate measures to assess the sensitivity of our results rather than to adjudicate among competing hypotheses. In addition, as a further robustness check, we calculate and analyze the share of each firm’s establishments or workers in each month that are in states with PSL mandates in effect, ignoring county-level mandates. This robustness check tests the possibility that state mandates may have more salience and influence than more local-level mandates.

Figure 2 displays the degree of variation in firm-level exposure to PSL mandates, taking the percentage of each firms’ workers who are employed at establishments in covered jurisdictions as the indicator. We see that across firms, this share ranges across nearly the entire distribution from 0% of some firms’ workers to nearly 95% of other firms’ workforce.

### Moderating variables

We construct two key moderating variables. The first is a measure of whether each firm’s headquarters is located in a place with a PSL mandate in effect. This measure is time varying as a function of the passage of PSL mandates, but we do not observe useful within-firm variation in headquarters locations (such as from moves). The second measure captures whether each firm engages in franchising. Drawing on legally mandated Franchise Disclosure Documents (FDD), we construct a dichotomous measure of if each firm franchises. None of the 135 firms in our data began or ended franchise operations over our observation period and so this is necessarily a time-invariant measure. We also construct a continuous measure of the share of each firms’ establishments that were franchised. To construct this measure, we divide the total number of franchised locations disclosed in each firm’s FDD by the sum of the total number of franchised locations and company-owned locations. This measure can vary based on the year of the FDD and on whether the figures from the start of the reporting year or end of the reporting year are used. However, because FDDs are not available to us in each year and because the range of variation over the observation period within firms is limited (Mean = .85, SD = .02), we average all available measures of the percent franchised by firm and treat this as a time-invariant measure.

### Analysis

We focus on respondents who are working in locations that are not covered by PSL mandates and, for these respondents, estimate the association between the degree of firm-level exposure to PSL mandates across its national business operations and respondent reports of being able to access PSL at work. We would find evidence of narrow compliance if there is no association between firm-level exposure to PSL mandates and respondents’ reports of access to PSL in places without PSL mandates in effect. In contrast, we would find evidence for intra-firm spillover if there is a positive association between firm-level exposure to PSL mandates and respondent reports of PSL access in non-covered locations.

To test these hypotheses, we estimate a series of linear probability models on the multiply imputed data. In Table 1, we limit the sample to workers in states or counties without PSL mandates and model the association between the firm-level exposure to PSL mandates and respondent’s report of PSL access. Our first set of models use the measure of firm-level exposure to PSL mandates as a percent of workers at the firm and then we substitute the measure of firm-level exposure as a percent of a firm’s establishments. In each case, we first estimate a model with worker-level demographic controls, then add job characteristics controls, then firm-level controls, then time-varying state-level controls, then state fixed-effects, and then year-by-month fixed-effects. We cluster standard errors at the state level.

We subject our main results to a series of robustness tests. First, we assess robustness to weighting the main results and to excluding observations collected in 2020 and in 2021, during the prime years of the COVID-19 Pandemic (Appendix Table A1). Second, we substitute our more restrictive measures of firm-level exposure to state PSL mandates, ignoring county-level mandates. We estimate the same set of models for the sample of workers who are employed in states without PSL mandates (Appendix Table A2). Third, three states—MI, NV, and ME—had paid time off laws that are distinct from paid sick time laws. In the main analyses, we treat these states as not having PSL. Their establishments/workers in these states are not counted as being exposed to PSL mandate and respondents in these states are considered to not be covered by PSL mandate. In Appendix Table A3, we report on the results of each alternative way of characterizing these states. For our measures of firm exposure, we estimate models that count establishments/workers in these states as being covered by PSL as well as models that omit these states from both the numerator and denominator of the exposure measures. For respondents, we estimate models that consider these states as having PSL and as not having PSL.

Fourth, we conduct falsification tests that substitute five fringe benefits that are conceptually distinct from paid sick leave in place of the paid sick leave dependent variable. The conceptual model underlying our research design is one in which firm-level exposure to PSL mandates may spillover within firms to non-covered locations. However, a competing hypothesis is that firms that are predisposed to take a high road approach to job conditions, including the provision of paid sick leave benefits, may be more inclined to operate in geographic locations with state or county PSL mandates. If this were the case, the estimated relationship between firm-level exposure and worker coverage in non-mandate locations could be spurious and driven by a selection process. If selectivity in a firm’s establishment locations with respect to high road employment practices explains the relationship between firm-level exposure to PSL mandates and workers’ coverage in non-mandate locations, we would also expect to see a similar pattern for other benefits. To operationalize this falsification test, we model the association between firm-level exposure to PSL mandates and workers’ reports of access to a set of six fringe benefits: PSL, as in our main models, and, separately, health insurance, dental, paid parental leave, retirement, and tuition benefits. We present the results of these falsification tests in Appendix Table A4 and Appendix Figure A1.

Our fifth and final robustness check tests for non-linearity in the relationship between PSL mandates and intra-firm spillover. Our main estimates model a linear association between a continuous measure of firm-level exposure to PSL mandates and the probability that a worker reports access to PSL in non-mandate workplaces. In separate analyses shown in Appendix Table A5 and Appendix Figure A2, we model firm-level exposure as a categorical variable, to capture non-linearities in the relationship. In particular, we were interested in whether there was evidence of a tipping point value for firm-level exposure to PSL mandates beyond which a relationship to PSL in non-mandated workplaces appeared. This separate analysis showed a pattern consistent with a dose-response relationship, with the strength of the association increasing when firm-level exposure rose above 15%.

In our final analyses, we extend the main results to test our hypotheses regarding moderation by measures of mandate salience and firm control. We estimate the extent to which the salience of PSL mandates, as proxied by a firms’ headquarters being in a place with a PSL mandate, moderates the key associations above. To do so, in Table 2, we re-estimate the main models in Table 1, including interactions between firm headquarters location and the measures of exposure to PSL mandates.^5^ We also estimate if these key associations are moderated by the degree of corporate control, proxied by firms’ franchise operations. In Table 3, we re-estimate the key models, first including an interaction between the measures of exposure to PSL mandates and a dichotomous measure of franchising and then between the measures of exposure to PSL mandates and a continuous measure of the percent of establishments that are franchisee-owned by firm. In our data, franchising is concentrated in the food service sector. It is possible then that any apparent franchise-effects are driven by food-service sector-specific dynamics. We re-estimate the moderation models above, restricting to the food service sector.

## RESULTS

Our key regression results appear in Table 1. We assess the association between a firm’s exposure to PSL mandates and workers’ reports of having access to paid sick leave at their job in non-covered workplace settings. We measure firm-level exposure to PSL mandates both in terms of the percent of the firm’s workers and the percent of the firm’s establishments subject to these mandates because of state or local legislation.

Table 1 shows the coefficients on these two measures across six model specifications that sequentially introduce additional controls and fixed-effects. Notably, the estimates change very little in magnitude or significance with the addition of these controls and fixed-effects. Model 6 shows our preferred, and most conservative specification, after adjusting for worker demographics, work characteristics, and state, year, month, and state-by-month fixed-effects. We find a coefficient of 0.005 (*p* < 0.01), which implies that for a 1 percentage point increase in the share of a firms’ workers who are covered by PSL mandates, the share of the firms’ workers who are not covered by mandates but still report access to PSL increases by 0.5 percentage points.

We find a substantive difference in the share of workers who report being able to access paid sick leave at their jobs, despite not being covered by PSL mandates, by the degree of their firm’s exposure to PSL mandates in other parts of the country. To size these associations, we plot predicted values from these models in Figure 3, using exposure defined as percent of workers on the left and percent of establishments on the right. We plot the predicted share of workers reporting access to PSL at firms with between 0% exposure to PSL mandates and 50% exposure (the 96th percentile of the firm-exposure distribution). While just over 25% of workers employed at firms with minimal exposure to PSL mandates (less than 5% of their workforce) report access to paid sick, 45% of workers employed at firms with significant exposure to PSL mandates (45% of their workforce) report access to paid sick leave—in places that do not mandate PSL.

### Robustness

In Appendix Table A1, we first reproduce our preferred model 6 from Table 1 in column 1. We then estimate sensitivity to excluding observations collected in 2020 and 2021, when the dynamics of the COVID-19 pandemic could have shaped PSL provision. We find no difference after removing these observations. In column 3, we assess the sensitivity of our results to weighting the estimates to correct for selection bias into the survey on demographics. The estimates are stable across this alternative specification.

In Appendix Table A2, we include a parallel set of model estimates which focus exclusively on the potential intra-firm spillover effects of state-level PSL mandates and ignore county-level mandates. These model estimates are nearly identical to those that model the potential intra-firm spillover of state or county-level PSL mandates across firm locations. Therefore, we find no evidence that local-level mandates operate differently or carry less influence than state-level mandates when it comes to intra-firm spillovers.

In Appendix Table A3, we see similar stability in the key estimates across various different approaches to treating the three states—MI, NV, and ME—that mandate paid time off, rather than Paid Sick Leave.

To test for the possibility that selectivity with respect to geographic location of firms could confound the relationship between firm-level exposure to PSL mandates and access to PSL benefits for workers in non-covered geographies, we present the results from falsification tests in Appendix Table A4 and Appendix Figure A1. In theory we expect access PSL benefits may diffuse across a company as firm-level exposure to mandates increases, but we have no reason to expect that other benefits would be associated with firm-level exposure to PSL mandates. In Appendix Table A4 and Appendix Figure A1 we see two noteworthy patterns. First, firm-level exposure to PSL mandates is associated with access to each type of fringe benefit. Second, the association between firm-level exposure to PSL mandates and worker access to PSL benefits in non-covered locations is about 3 times larger in magnitude than the association with any of five other fringe benefits. These findings suggest that there may be some selectivity in the geographies in which firms choose to operate, and that this may explain a portion of the association between firm-level exposure to PSL mandates and worker access to PSL benefits in non-covered locations. However, the majority of this association is not explained by selection and is consistent with the theoretical intra-firm spillovers of PSL coverage as firm-level exposure to mandates increases.

As a final robustness check, we test for potential non-linearities in the association between firm-level exposure to PSL mandate and access to PSL benefits in non-covered workplaces. Our main estimates model the association between a continuous measure of firm-level exposure. In Appendix Table A5, we substitute a categorical measure of firm-level exposure. This categorical measure compares firms with less than 15% of their workers or establishments covered by state or county PSL mandates to firms with 15% to 30%, 30% to 45%, or greater than 45%. These alternative results, plotted in Appendix Figure A2, show that the relationship between firm-level exposure and access to PSL benefits in non-covered locations follows a dose-response, approximately linear relationship.

### Moderating forces

We hypothesize that the association between firm-level exposure to PSL mandates in their business operations and PSL access for their workers in places where not mandated, may be stronger when the salience of PSL mandates is higher. In Table 2, we show the results from a set of models that test if a firm’s headquarters being located in a place with a PSL mandate moderates the association between firm exposure to PSL mandates and the share of workers who report access to PSL where not legally mandated. The table follows a similar layout to Table 1, displaying estimates using both the share of a firm’s workers and the share of a firm’s establishments exposed to PSL mandates, defined by state or county mandate, as predictors of the PSL access of workers in non-PSL states or counties.

Across all model specifications, the association between PSL mandate exposure and PSL access was significantly stronger for firms that were head-quartered in places with PSL mandates (ranging from B = 0.004, *p* < 0.001 to B = 0.005, *p* < 0.001). On average, the association between the percentage of employer establishments or its labor force subject to PSL mandates and PSL coverage for workers not subject to mandates is amplified by about half a percentage point when companies are headquartered in a place with a PSL mandate in place. In Appendix Figure 3, we plot the predicted values from the top panel of Table 2. The plot shows that the relationship between firm-level exposure to PSL mandates is stronger (steeper sloped) when a firm is headquartered in a place with a PSL mandate. When firm-level exposure rises to 50%—meaning that 50% of a firm’s workforce is covered by a state or local PSL mandate—there is a sizeable gap in the PSL benefits of workers in non-covered locations depending on whether firms are headquartered in a PSL-mandate location or not. In particular, at this level of firm exposure to PSL mandates, 50% of workers in workplace locations with a PSL mandate nevertheless report having PSL benefits when the firm headquarters is in a covered location, compared to fewer than 40% of their counterparts employed by firms headquartered outside of covered locations.

In Table 3, we examine the degree to which corporate control over establishments, captured here by franchisee structure, shapes the key associations of interest. The key coefficients in this table are the interactions between whether the firm franchises and the share of workers/establishments exposed to PSL mandates and the interactions between the share of a firms’ establishments that are franchised and the share of workers/establishments exposed to PSL mandates. For each model, we estimate a version that includes all firms (column 1) and a version limited to fast food firms (column 2).

We find consistent evidence that the share of a firm’s workers/establishments covered by PSL has a much weaker influence on whether non-covered workers report access to PSL when the firm engages in franchising behavior (column 1). Given that franchising is most common in the fast-food sector, the weaker relationship between firm exposure to PSL mandates and PSL coverage for workers in non-mandate locations among firms that engage in franchising could be an artifact of low road practices in the fast-food sector. However, rather than simply being an artifact of practices in the food service sector, this moderation is stronger when we compare franchised against non-franchised food service firms (column 2). We plot the predicted values from the top panel of Table 3 in Appendix Figure A4. We see that for all industries and for the fast-food sector, that non-franchised firms exhibit a stronger pattern of intra-firm spillover compared with franchised firms. This contrast is particularly stark in the fast-food sector, where franchised firms exhibit little spillover of PSL benefits to workers in non-covered locations while, among non-franchised fast-food firms, PSL spillovers to workers in non-covered locations increases dramatically with firm-level exposure to PSL mandates.

## DISCUSSION

We take advantage of unique employer-employee matched data collected by the Shift Project to examine if state and local PSL mandates have intra-firm spillovers by which PSL is extended to workers in non-covered jurisdictions. We find robust evidence that is consistent with such intra-firm spillover. As the share of a company’s workforce or establishments that are subject to paid sick leave mandates rises, so too does access to paid sick leave increase for the broader workforce, employed in non-covered localities. This association is sizable in magnitude: Moving from low exposure to mandates to high exposure to mandates is associated with an increase in voluntary coverage of workers from 25% to 45% at establishments not covered by paid sick leave mandates. We also subject our findings to a falsification check—showing that while PSL access for non-covered workers increases with company exposure to PSL laws, that is not the case for other employer-provided benefits. Further, we show that, as hypothesized, the association between firm-level exposure to PSL mandates and the intra-firm spillover of coverage to workers in non-covered locations is amplified when corporate headquarters are located in a place with a PSL mandate in place. We speculate that the policy setting where corporate headquarters are located may raise the salience of PSL benefits, normalize the provision of these benefits, and create some corporate impetus for extending the benefit to workers nationally, even when not legally required to do so. Further, we show that franchised firms exhibit less intra-firm spillover compared with non-franchised firms. This was especially so in the fast-food sector, where franchising is prevalent. This finding reveals an inherent limitation to intra-firm spillovers imposed when company policies and practices become decentralized and locally-determined because of franchising.

Prior literature has documented the presence of spatial spillovers from sub-national minimum wage standards. We advance the literature on labor standards spillovers by providing evidence of a distinct process, one of intra-firm spillover in which multi-state firms with partial exposure to state and local PSL standards are more likely to offer PSL to uncovered workers. Our results suggest that when cities and states take action to mandate labor standards in their jurisdiction, they may also effectively shape firm behavior beyond their borders.

It is also possible that such intra-firm spillovers could catalyze broader effects. One possibility is the intra-firm spillovers that lead multi-state firms to enhance their benefits in non-covered jurisdictions may induce other local firms in the geographic areas without PSL laws to expand their offerings. Another is that multi-state firms might advocate for paid sick leave in states without such labor standards in order to even the playing field with local competitors who do not face intra-firm spillovers.

Our results also have implications for studies that attempt to estimate the effects of PSL mandates in covered jurisdictions. Prior work (such as Callison & Pesko, 2022, & Schneider, 2020) which uses difference-in-difference methods may underestimate the effects of PSL standards due to leakage to control units via intra-firm spillovers. There is not an easy solution to such threats as few datasets identify employer and focusing only on small firms (which may have less multi-state exposure) risks generalizability.

Our research is subject to three limitations that should be kept in mind when interpreting results. First, the Shift Project survey is a non-probability sample, and workers who respond to Shift Project surveys may differ from the broader population of service sector workers. To adjust for biases on observed demographic characteristics we weight our sample to align with the characteristics of workers in the American Community Survey. Because our research design is comparative—examining differences in paid sick leave coverage for workers whose employers vary in their exposure to paid sick leave mandates—remaining biases are likely to net out and be less concerning. We can think of no compelling reason why sample selection biases would differentially affect workers whose employers had higher and lower exposure to paid sick leave mandates.

Second, our dependent variable is a measure of paid sick leave benefits that is self-reported by workers and may not precisely correspond to firm provision of PSL benefits, because of incomplete or inaccurate worker knowledge of employer-provided fringe benefits. Our measure of paid sick leave benefits should therefore be strictly interpreted as workers’ perception that PSL benefits are available rather than as an indicator of company policy, though we expect worker perception and company policy to be strongly correlated. Further, from the standpoint of practical significance, we would argue that workers’ perceptions that they have access to PSL benefits is an important prerequisite to using these benefits.

Third, a threat to our conclusions is the selection process through which firms choose their establishment locations. If high-road employers are more amenable to locating their business in the more stringent regulatory environments, then the association between exposure to paid sick leave mandates and extension of these benefits to non-covered workers could be driven in part by unobserved tendencies toward high-road labor practices. Because most of the variation in firm exposure to PSL mandates is between-firm rather than within firms over time, we are not able to include firm fixed effects to control for unobserved attributes of firms that may confound our estimated relationships. Instead, to gauge the presence of this type of selectivity, we substituted one of a set of five fringe benefits in place of PSL benefits as the outcome in our models. These tests confirmed that selection plays a role in the association between firm-level exposure to PSL mandates and PSL coverage for workers in non-mandate locations, but that most of this association is not attributable to selection.

We set out in this paper to explore the role that firms play as a mechanism through which local labor standards may diffuse across the nation. Although firms face pressure to minimize labor costs and often privilege cost-cutting considerations over worker well-being, job satisfaction, and worker retention, we nevertheless find robust and consistent evidence that firm-level exposure to paid sick leave mandates is associated with voluntary expansion of paid sick leave coverage beyond the narrow locations in which it is legally required. These patterns show that local policies can spur broader voluntary action on the part of companies that leads to better working conditions for the broader workforce.

## Supplementary Material

supplementary material

Additional supporting information can be found online in the Supporting Information section at the end of this article.

## Figures and Tables

**FIGURE 1:**
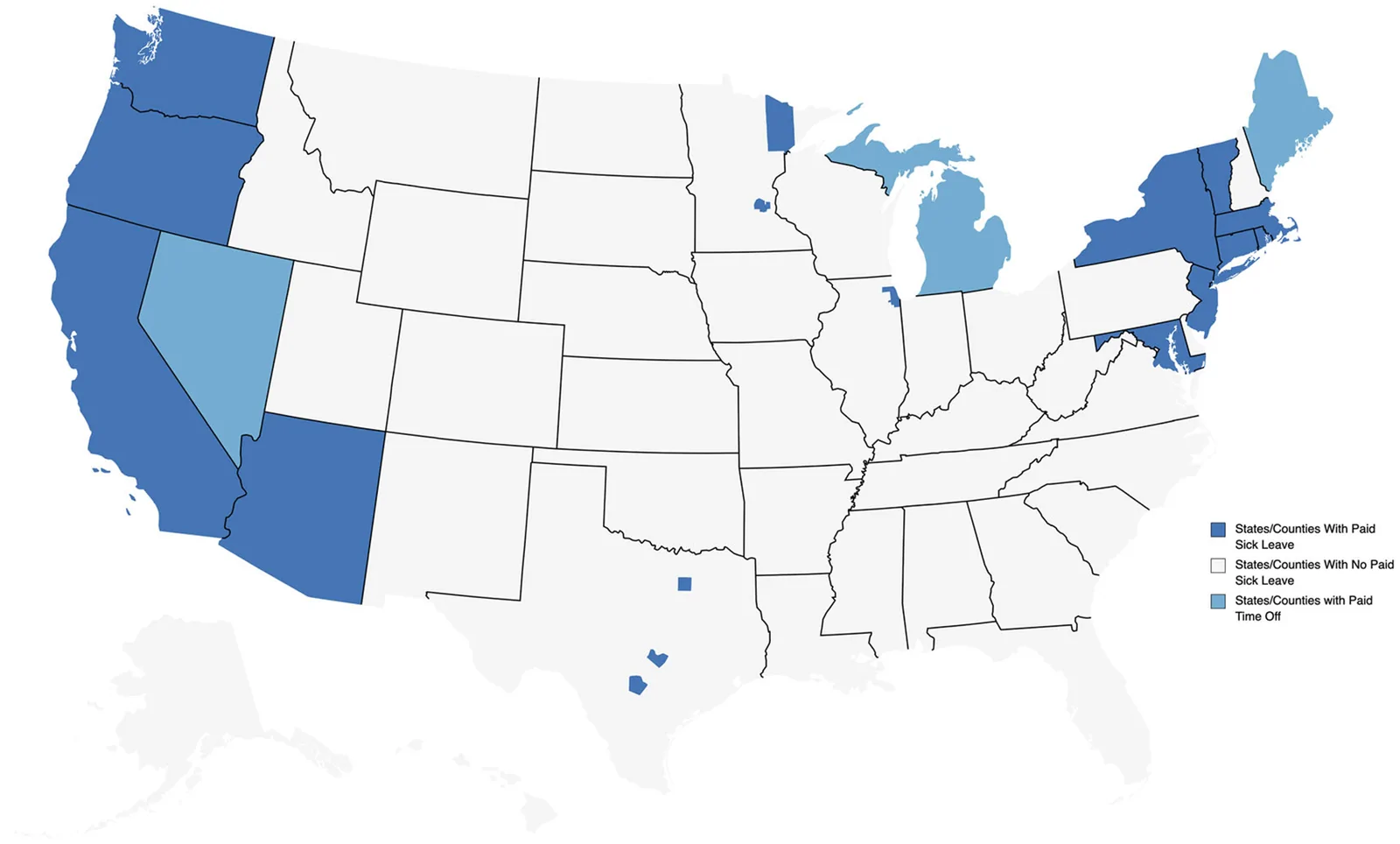
States with paid sick leave laws, 2021. *Source*: “Interactive Overview of Paid Sick Time Laws in the United States” (A Better Balance, n.d.). *Note*: In March of 2021, the PSL mandates in Austin, Dallas, and San Antonio, Texas, were deemed to be preempted by a state law and consequently were struck down.

**FIGURE 2:**
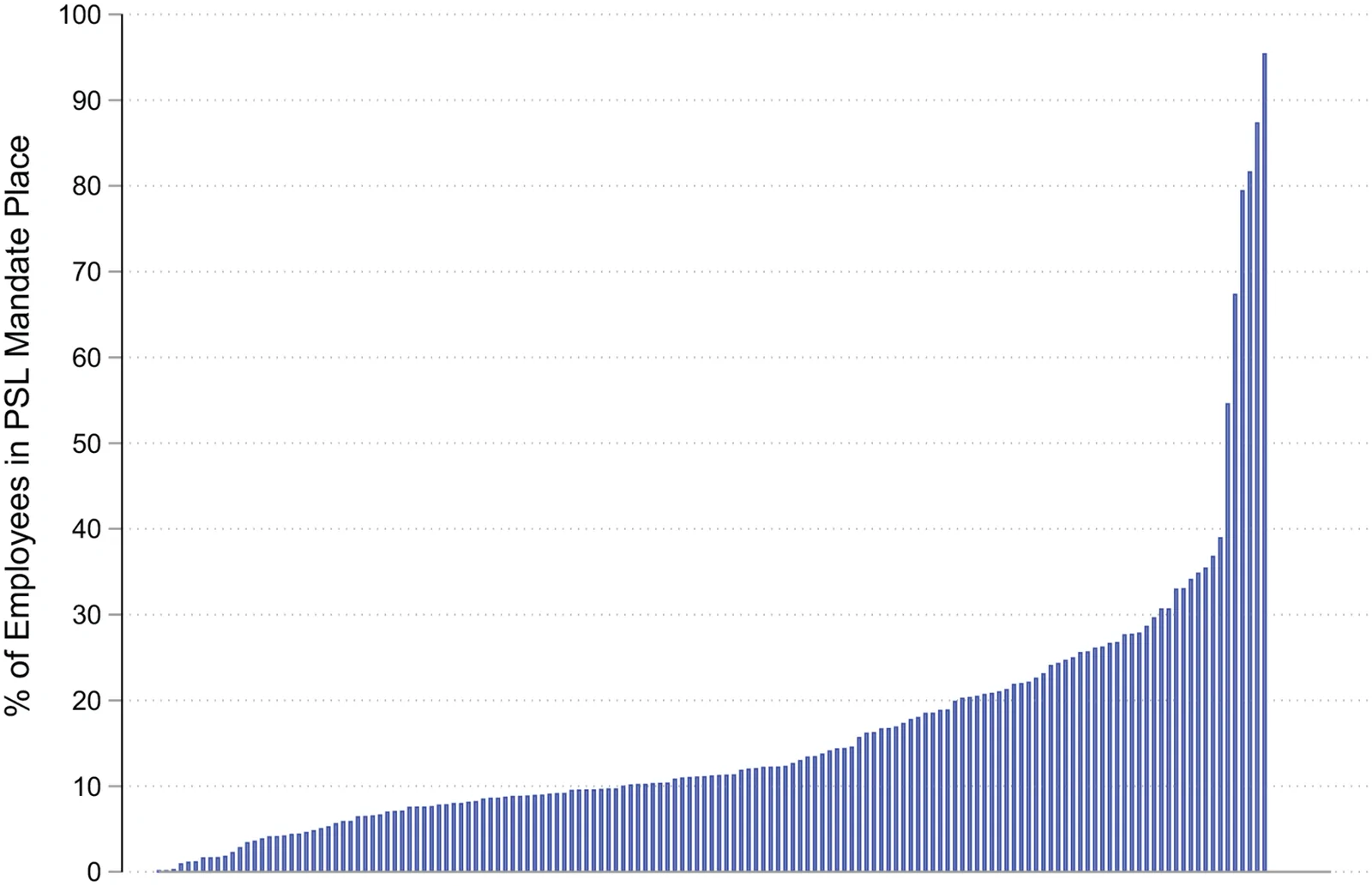
Percentage of a firm’s workers covered by state or county PSL mandates, 2020 by firm. *Source*: National Partnership for Women and Families (PSL mandate data) & Reference USA (employee/establishment data). *Notes*: Each bar represents one firm. Shares of workers in PSL mandate places are calculated at the firm-county-month level, then averaged at the year level (2020 only).

**FIGURE 3:**
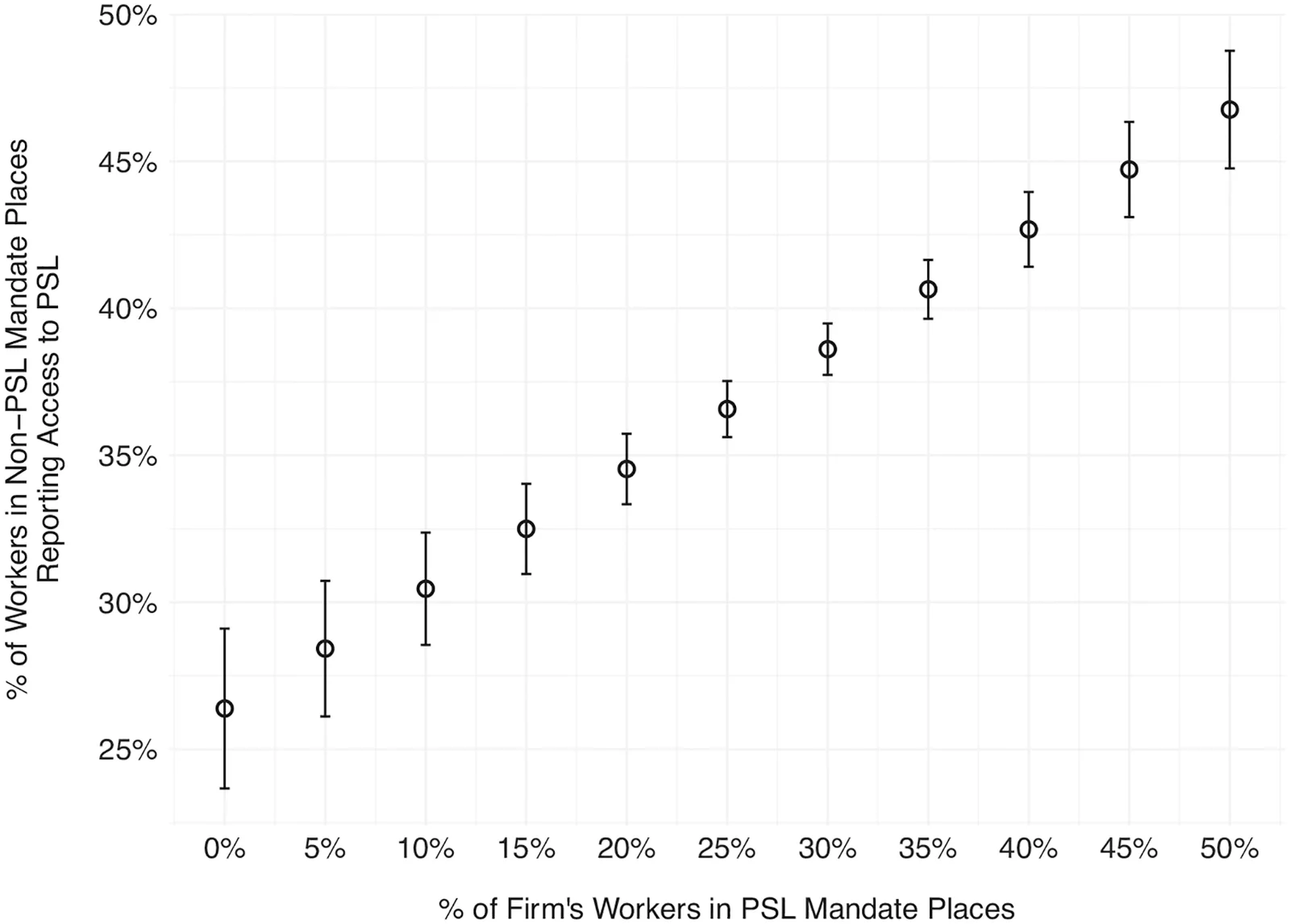
Predicted share of workers in non-PSL mandate places reporting access to Paid Sick Leave, by firm exposure to PSL mandates. *Source*: Shift Project data, 2018 to 2021. *Notes*: Predicted values from linear regression model, with the percentage of firm’s workers in a PSL mandate place as a continuous regressor. Demographic controls: gender, race, age, parental status, speaking a non-English language at home, being enrolled in school, cohabitation status. Work controls: hourly wage, usual weekly hours, manager status, union membership, job tenure. Also includes state and year/month/year*month fixed effects. Robust standard errors clustered at state level.

**TABLE 1:** Association between firm-level exposure to PSL mandates and workers’ reports of access to PSL in non-PSL mandate places.

	Workers in non-PSL states or counties					
	M1	M2	M3	M4	M5	M6
*Share in PSL covered state or county*						
Percent of workers	0.006***	0.004***	0.005***	0.005***	0.005***	0.005***
Percent of establishments	0.006***	0.004***	0.005***	0.005***	0.005***	0.005***
Demographic controls	✓	✓	✓	✓	✓	✓
Work controls		✓	✓	✓	✓	✓
Firm controls			✓	✓	✓	✓
State controls				✓	✓	✓
State FE					✓	✓
Year, Month, Year x Month FE						✓
*N*	29381	29381	29381	29381	29381	29381

*Source*: Shift Project data, 2018 to 2021.

*Notes*: Models regress reported access to PSL among workers in states or counties without PSL mandates on the percent of workers/establishments at the same firm who are in states or counties with PSL mandates. Demographic controls: gender, race, age, parental status, speaking a non-English language at home, being enrolled in school, cohabitation status. Work controls: hourly wage, usual weekly hours, manager status, union membership, job tenure. Firm controls: industry, total number of employees (by month), total number of establishments (by month). State controls: unemployment rate, minimum wage, union membership, breakdowns of age/gender/race/education. Robust standard errors clustered at state level.

+ *p* < 0.10,

* *p* < 0,05,

** *p* < 0.01,

*** *p* < 0.001

**TABLE 2:** Moderation of association between firm-level exposure to PSL mandates and workers’ reports of access to PSL in non-mandate places—First headquarters location.

	PSL access in non-mandate places
*Share in PSL covered state or county*	
Percent of workers	−0.001**
Firm headquartered in PSL mandated place	−0.046
Percent of workers covered by PSL x HQ covered	0.004***
*Share in PSL covered state or county*	
Percent of establishments	0.002**
Firm headquartered in PSL mandated place	−0.059^+^
Percent of establishments covered by PSL x HQ covered	0.005***
Observations	29381

*Source*: Shift Project data, 2018 to 2021.

*Notes*: Dependent variable: reported access to PSL among workers in states/counties not covered by PSL mandates. All models include demographic controls, work controls, firm controls, state controls, state fixed effects, and year/month/year*month fixed effects. Demographic controls: gender, race, age, parental status, speaking a non-English language at home, being enrolled in school, cohabitation status. Work controls: hourly wage, usual weekly hours, manager status, union membership, job tenure. Firm controls: industry, total number of employees (by month), total number of establishments (by month). State controls: unemployment rate, minimum wage, union membership, breakdowns of age/gender/race/education. Robust standard errors clustered at state level.

+ *p* < 0.10,

* *p* < 0.05,

** *p* < 0.01,

*** *p* < 0.001

**TABLE 3:** Moderation of association between firm-level exposure to PSL mandates and workers’ reports of access to PSL in non-PSL mandate places—Firm franchising.

	PSL access in non-mandate places	
	All industries	Food industry
*Share in a PSL covered state or county*		
Percent of workers covered by PSL	0.005***	0.012**
Franchisor firm	0.129***	0.081**
Percent of workers covered by PSL x Franchisor firm	−0.005***	−0.012***
*Share in a PSL covered state or county*		
Percent of establishments covered by PSL	0.005***	0.012***
Franchisor firm	−0.130***	0.081**
Percent of establishments covered by PSL x Franchisor firm	−0.005***	−0.012***
*Share in a PSL Covered State or County*		
Percent of workers covered by PSL	0.005***	0.012***
Percent franchised	−0.117***	0.108***
Percent of firm’s workers covered by PSL x Percent franchised	−0.006***	−0.013***
*Share in a PSL Covered State or County*		
Percent of establishments covered by PSL	0.005***	0.012***
Percent franchised	−0.125***	0.107***
Percent of a firm’s establishments covered by PSL x Percent franchised	−0.005***	−0.014***
*N*	29481	11272

*Source*: Shift Project data, 2018 to 2021.

*Notes*: Dependent variable: reported access to PSL among workers in states/counties not covered by PSL mandates. “Franchisor firm” = binary indicator. All models include demographic controls, work controls, firm controls, state controls, state fixed effects, and year/month/year*month fixed effects. Demographic controls: gender, race, age, parental status, speaking a non-English language at home, being enrolled in school, cohabitation status. Work controls: hourly wage, usual weekly hours, manager status, union membership, job tenure. Firm controls: industry, number of employees (by month), total number of establishments (by month). State controls: unemployment rate, minimum wage, union membership, breakdowns of age/gender/race/education. Robust standard errors clustered at state level.

+ *p* < 0.10,

* *p* < 0.05,

** *p* < 0.01,

*** *p* < 0.001

## Data Availability

The data that support the findings of this study are openly available in Dataverse depository: https://dataverse.harvard.edu/dataset.xhtml?persistentId=doi:10.7910/DVN/X8JM22.

## Footnotes

1 We exclude Waves 1 and 2 of the data because the Shift Project did not ask respondents about access to paid sick leave in those waves.

2 Three states—Michigan, Nevada, and Maine—do not have PSL mandates, but do have a more general provision for paid time off. In our main models, we treat these states as not having PSL mandates. However, as shown in Appendix Table A1, our results are robust to various different ways of treating these states—including as having PSL mandates and as omitted from both the numerator and denominator.

3 All appendices are available at the end of this article as it appears in JPAM online. Go to the publisher’s website and use the search engine to locate the article at https://onlinelibrary.wiley.com

4 This measure has been deployed in a number of prior papers, which also serve as a useful validation check. Overall, approximately 50% of workers in the Shift data report access to paid sick leave (Harknett & Schneider, 2022) with substantial between-firm variation (Schneider & Harknett, 2020b). Prior research has shown that this measure effectively captures variation induced by PSL mandates. Across states, access to PSL was significantly higher (68%) in states with mandates as compared to states without (40%; Harknett & Schneider, 2022). Further, the measure also captured changes over time in mandated PSL access, detecting a 28 percentage point increase in access to PSL among WA-state workers in a difference-in-difference analysis as a result of the implementation of WA PSL law (Schneider, 2020). The measure also effectively detected a company-level change, finding that PSL access among Olive Garden workers increased from 23% to 66% in a difference-in-difference model following the company’s voluntary provision of PSL in 2020 (Schneider et al., 2021).

5 We assess robustness to excluding respondents at IKEA, the one firm in our data with an overseas headquarters location and find no substantive changes to our estimates (Appendix Table A7 and Appendix Figure A5).

## References

[R1] A Better Balance. (n.d.). Interactive overview of paid sick time laws in the United States. https://www.abetterbalance.org/paid-sick-time-laws/

[R2] A Better Balance. (2024). Local paid sick time laws that are no longer in effect. https://www.abetterbalance.org/resources/new-jersey-local-paid-sick-time-laws-now-preempted/

[R3] Alexander C, & Haley-Lock A. (2015). Underwork, work-hour insecurity, and a new approach to wage and hour regulation. Industrial Relations: A Journal of Economy and Society, 54(4). 10.1111/irel.12111

[R4] Appelbaum E, Bernhardt A, & Murnane RJ. (2003). Low-wage America: How employers are reshaping opportunity in the workplace. Russell Sage Foundation.

[R5] Baker D. (2020). CORRECTION: This is what minimum wage would be if it kept pace with productivity. Center for Economic and Policy Research. https://cepr.net/this-is-what-minimum-wage-would-be-if-it-kept-pace-with-productivity/

[R6] Berger DW, Herkenhoff KF, & Mongey S. (2019). Labor market power [Working paper no. 25719]. National Bureau of Economic Research. https://www.nber.org/system/files/working_papers/w25719/revisions/w25719.rev0.pdf

[R7] Bernhardt A, Spiller MW, & Polson D. (2013). All work and no pay: Violations of employment and labor laws in Chicago, Los Angeles and New York City. Social Forces, 91(3), 725–746. 10.1093/sf/sos193

[R8] Callison K, & Pesko MF. (2022). The effect of paid sick leave mandates on coverage, work absences, and presenteeism. Journal of Human Resources, 57(4), 1178–1208. 10.3368/jhr.57.4.1017-9124r2 PMC9266646 35812986

[R9] Callison K, Pesko MF, Phillips S, & Sosa JA. (2023). Cancer screening after the adoption of paid-sick-leave mandates. New England Journal of Medicine, 388(9), 824–832. 10.1056/NEJMsa2209197 PMC10084522 36856618

[R10] Card D, Mas A, Moretti E, & Saez E. (2012). Inequality at work: The effect of peer salaries on job satisfaction. American Economic Review, 102(6), 2981–3003. 10.1257/aer.102.6.2981

[R11] Carré F, & Tilly C. (2017). Where bad jobs are better: Retail jobs across countries and companies. Russell Sage Foundation.

[R12] Cengiz D, Dube A, Lindner A, & Zipperer B. (2019). The effect of minimum wages on low-wage jobs. Quarterly Journal of Economics, 134(3), 1405–1454. 10.1093/qje/qjz014

[R13] Clemens J. (2021). How do firms respond to minimum wage increases? Understanding the relevance of non-employment margins. Journal of Economics Perspectives, 35(1), 51–72. 10.1257/jep.35.1.51

[R14] Clemens J, & Strain M. (2020). Understanding “wage theft”: Evasion and avoidance responses to minimum wage increases [Working paper no. 26969]. National Bureau of Economic Research. https://www.nber.org/papers/w26969

[R15] Colla CH, Dow W. h., Dube A, & Lovell V. (2014). Early effects of the San Francisco paid sick leave policy. American Journal of Public Health, 104(12), 2453–2460. 10.2105/AJPH.2013.301575 PMC4232165 24432927

[R16] de Bruin WB, Saw H-W, & Goldman DP. (2020). Political polarization in U.S. residents’ COVID-19 risk perceptions, policy preferences, and protective behaviors. Journal of Risk and Uncertainty, 61(2), 177–194. 10.1007/s11166-020-09336-3 PMC7672261 33223612

[R17] Deputy. (2022). Navigate fair workweek laws and protect your business. https://www.deputy.com/fair-workweek

[R18] Derenoncourt E, & Weil D. (2025). Voluntary minimum wages: The local labor market effects of national retailer policies. Quarterly Journal of Economics. 10.1093/qje/qjaf016

[R19] DeRigne L, Stoddard-Dare P, & Quinn L. (2016). Workers without paid sick leave less likely to take time off for illness or injury compared to those with paid sick leave. Health Affairs, 35(3), 520–527. 10.1377/hlthaff.2015.0965 26953308

[R20] Deschenaux J. (2019, May 31). How to comply with new state and local laws. HR Quarterly. https://www.shrm.org/topics-tools/news/hr-magazine/how-to-comply-new-state-local-laws

[R21] Dharmasanker S, & Yoo H. (2023). Assessing the main and spillover effects of Seattle’s minimum wage on establishment decisions. Regional Science and Urban Economics, 99(C). 10.1016/j.regsciurbeco.2022.103865

[R22] Dube A, & Lindner AS. (2024). Minimum wages in the 21st century [Working paper 32989]. National Bureau of Economic Research. 10.3386/w32878

[R23] Elmore A, & Griffith KL. (2021). Franchisor power as employment control. California Law Review, 109(4), 1317–1371. 10.15779/Z38QJ77Z9J

[R24] Endenich C, Brandau M, & Hoffjan A. (2011). Two decades of research on comparative management accounting—Achievements and future directions. Australian Accounting Review, 21(4), 365–382. 10.1111/j.1835-2561.2011.00149.x

[R25] Galvin DJ. (2016). Deterring wage theft: Alt-labor, state politics, and the policy determinants of minimum wage compliance. Perspectives on Politics, 14(2), 324–350. 10.1017/S1537592716000050

[R26] Galvin DJ. (2019). From labor law to employment law: The changing politics of workers’ rights. Studies in American Political Development, 33(1), 50–86. 10.1017/S0898588X19000038

[R27] Gerken HK, & Holtzblatt A. (2014). The political safeguards of horizontal federalism. Michigan Law Review, 113, 1. https://repository.law.umich.edu/cgi/viewcontent.cgi?article=1181&context=mlr

[R28] Gould E, & Schieder J. (2017). Work sick or lose pay? The high cost of being sick when you don’t get paid sick days. Economic Policy Institute. https://www.epi.org/publication/work-sick-or-lose-pay-the-high-cost-of-being-sick-when-you-dont-get-paid-sick-days/

[R29] Hacker JS. (2006). The great risk shift: The assault on American jobs, families, health care, and retirement, and how you can fight back. Oxford University Press.

[R30] Harknett K, & Schneider D. (2022). Mandates narrow gender gaps in paid sick leave coverage for low-wage workers in the US. Health Affairs, 41(11), 1575–1582. 10.1377/hlthaff.2022.00727 PMC10700023 36343314

[R31] Harknett K, Schneider D, & Irwin V. (2021). Improving health and economic security by reducing work schedule uncertainty. Proceedings of the National Academy of Sciences, 118(42). 10.1073/pnas.2107828118 PMC8545454 34625472

[R32] Hazell J, Patterson C, Sarsons H, & Taska B. (2022). National wage setting [Working paper no. 30623]. National Bureau of Economic Research. 10.3386/w30623

[R33] Heymann J, Rho HJ, Schmitt J, & Earle A. (2009). Contagion nation: A comparison of paid sick day policies in 22 countries. Center for Economic and Policy Research. https://www.cepr.net/documents/publications/paid-sick-days-2009-05.pdf

[R34] Heymann J, Sprague A, Earle A, McCormack M, Waisath W, & Raub A. (2021). US sick leave in global context: US eligibility rules widen inequalities despite readily available solutions. Health Affairs, 40(9), 1501–1509. 10.1377/hlthaff.2021.00731 34310189

[R35] Jardim E, Long MC, Plotnick R, Vigdor J, & Wiles E. (2024). Local minimum wage laws, boundary discontinuity methods, and policy spillovers. Journal of Public Economics, 234. 10.1016/j.jpubeco.2024.105131

[R36] Jencks C, Smith M, Acland H, Bane MJ, Cohen D, Gintis H, Heynes B, & Michelson S. (1972). Inequality: A reassessment of the effect of family and schooling in America. Basic Books.

[R37] Ji M, & Weil D. (2015). The impact of franchising on labor standards compliance. ILR Review, 68(5), 977–1006. 10.1177/0019793915586384

[R38] Kalleberg AL. (2011). Good jobs, bad jobs: The rise of polarized and precarious employment systems in the United States, 1970s to 2000s. Russell Sage Foundation.

[R39] Kattih N, Mansour F, & Mixon FG. (2021). Keeping what you like: Grandfathering and health insurance coverage take-up rates under the ACA. Journal of Economics and Finance, 45(1), 188–199. 10.1007/s12197-020-09530-8

[R40] Lakhani T. (2022). How and why does franchise ownership affect human resource practices? Evidence from the US hotel industry. ILR Review, 75(2), 321–347. 10.1177/0019793920972661

[R41] Lambert SJ. (2008). Passing the buck: Labor flexibility practices that transfer risk onto hourly workers. Human Relations, 61(9), 1203–1227.

[R42] Lambert SJ, & Haley A. (2021). Implementing work scheduling regulation: Compliance and enforcement challenges at the local level. ILR Review, 74(5), 1231–1257. 10.1177/00197939211031227

[R43] Lovell V. (2004). No time to be sick: Why everyone suffers when workers don’t have paid sick leave. IWPR. https://iwpr.org/iwpr-general/no-time-to-be-sick-why-everyone-suffers-when-workers-dont-have-paid-sick-leave/

[R44] Maclean JC, Pichler S, & Ziebarth NR. (2020). Mandated sick pay: Coverage, utilization, and crowding-in [Working paper no. 26832]. National Bureau of Economic Research. 10.3386/w26832 PMC12510734 41078455

[R45] McKinnish T. (2017). Cross-state differences in the minimum wage and out-of-state commuting by low-wage workers. Regional Science and Urban Economics, 64, 137–147. 10.1016/j.regsciurbeco.2017.02.006

[R46] Merton RK, & Rossi A. (1957). Social theory and social structure. Free Press.

[R47] Mulligan CB. (2020). The employer penalty, voluntary compliance, and the size distribution of firms: Evidence from a survey of small businesses. Tax Policy and the Economy, 34, 139–171. 10.1086/708173

[R48] National Partnership for Women & Families. (n.d.). Paid sick days. https://nationalpartnership.org/economic-justice/paid-sick-days/

[R49] National Partnership for Women & Families. (2023, October). Paid sick days statutes.

[R50] Paid Sick Leave Laws [Webinar]. (2022). https://paidsickleavelaws.splashthat.com/%E2%80%9C

[R51] Palladino L. (2021). Financialization at work: Shareholder primacy and stagnant wages in the United States. Competition & Change, 25(3–4), 382–400. 10.1177/1024529420934641

[R52] Petrucci L, Loustaunau L, Scott E, & Stepick L. (2021). Persistent unpredictability: Analyzing experiences with the first statewide scheduling legislation in Oregon. ILR Review. 10.1177/00197939211064902

[R53] Pew Research Center. (2014, June 12). Political polarization in the American public: How increasing ideological uniformity and partisan antipathy affect politics, compromise and everyday life. https://www.pewresearch.org/politics/2014/06/12/political-polarization-in-the-american-public/

[R54] Pichler S, & Ziebarth NR. (2017). The pros and cons of sick pay schemes: Testing for contagious presenteeism and noncontagious absenteeism behavior. Journal of Public Economics, 156, 14–33. 10.1016/j.jpubeco.2017.07.003

[R55] Pichler S, Wen K, & Ziebarth NR. (2020). COVID-19 emergency sick leave has helped flatten the curve in the United States. Health Affairs, 39(12), 2197–2204. 10.1377/hlthaff.2020.00863 33058691

[R56] Pichler S, Wen K, & Ziebarth NR. (2021). Positive health externalities of mandating paid sick leave. Journal of Policy Analysis and Management, 40(3), 715–743. 10.1002/pam.22284

[R57] Pomeranz JL, Silver D, Lieff SA, & Pagán JA. (2022). State paid sick leave and paid sick-leave preemption laws across 50 U.S. states, 2009–2020. American Journal of Preventive Medicine. 10.1016/j.amepre.2021.11.018 35459452

[R58] Raeburn NC. (2004). Changing corporate America from inside out: Lesbian and gay workplace rights (Vol. 20, New ed.). University of Minnesota Press.

[R59] Reich M, Jacobs K, & Dietz M. (Eds.). (2014). Requiring equal benefits for domestic partners. In When mandates work: Raising labor standards at the local level (pp. 156–196). University of California Press.

[R60] Romich JL. (2017). Local mandate improves equity of paid sick leave coverage: Seattle’s experience. BMC Public Health, 17(1). 10.1186/s12889-016-3925-9 PMC5225554 28077115

[R61] Ross M, & Bateman N. (2019). Meet the low-wage workforce. Brookings. https://www.brookings.edu/research/meet-the-low-wage-workforce/

[R62] Schneider D. (2020). Paid sick leave in Washington State: Evidence on employee outcomes, 2016–2018. American Journal of Public Health, 110(4), 499–504. 10.2105/AJPH.2019.305481 PMC7067115 32078341

[R63] Schneider D, & Harknett K. (2019). Consequences of routine work-schedule instability for worker health and well-being. American Sociological Review, 84(1), 82–114. 10.1177/0003122418823184 PMC7730535 33311716

[R64] Schneider D, & Harknett K. (2020a). Essential and vulnerable: Service-sector workers and paid sick leave. The Shift Project.

[R65] Schneider D, & Harknett K. (2020b). Estimates of workers who lack access to paid sick leave at 91 large service sector employers. The Shift Project. https://shift.hks.harvard.edu/paid-sick-leave-brief/

[R66] Schneider D, & Harknett K. (2022). What’s to like? Facebook as a tool for survey data collection. Sociological Methods & Research, 51(8), 108–140. 10.1177/0049124119882477 PMC9957582 36845408

[R67] Schneider D, Harknett K, & Vivas-Portillo E. (2021). Olive Garden’s expansion of paid sick leave during COVID-19 reduced the share of employees working while sick. Health Affairs, 40(8), 1328–1336. 10.1377/hlthaff.2020.02320 PMC9118176 34339244

[R68] Slopen M. (2023). The impact of paid sick leave mandates on women’s health. Social Science & Medicine. 10.1016/j.socscimed.2023.115839 36989657

[R69] Slopen M. (2024). The impact of paid sick leave mandates on women’s employment and economic security. Journal of Policy Analysis and Management, 43(3). 10.1002/pam.22582

[R70] Stansbury A. (2021). Employer concentration suppresses wages for several million U.S. workers: Antitrust and labor market regulators should respond. Equitable Growth. https://equitablegrowth.org/employer-concentration-suppresses-wages-for-several-million-u-s-workers-antitrust-and-labor-market-regulators-should-respond/

[R71] Storer A. (2022). Workplace stratification of scheduling practices as a source of social comparison. Academy of Management Proceedings, 2022(1). 10.5465/AMBPP.2022.54

[R72] Stouffer SA, Suchman EA, DeVinney LC, Star SA, & Williams RM Jr. (1949). The American soldier: Adjustment during Army life. Princeton University Press.

[R73] Tsoukalas A, Galvin DJ, Fine J, & Round J. (2021, March). Florida policymakers need to reassess how the minimum wage is enforced. Rutgers School of Management and Labor Relations. https://cdn.prod.website-files.com/5cd5801dfdf7e5927800fb7f/632fb6e3d183d2079669024e_RT_Wage_Theft_FINAL.pdf

[R74] Ton Z. (2014). The good jobs strategy: how the smartest companies invest in employees to lower costs and boost profits. Houghton Mifflin Harcourt.

[R75] Veblen T. (1899). The theory of the leisure class. Macmillan Publishers.

[R76] Wage and Hour Division. (2014, November 6). Workers face millions in unpaid wages in southern California garment industry [Press release]. U.S. Department of Labor. https://www.dol.gov/newsroom/releases/whd/whd20141106

[R77] Weil D. (2014). The fissured workplace: Why work became so bad for so many and what can be done to improve it. Harvard University Press.

[R78] Williams JC, Lambert SJ, Kesavan S, Fugiel PJ, Ospina LA, Rapoport ED, Jarpe M, Bellisle D, Pendem P, McCorkell L, & Adler-Milstein S. (2018). Stable scheduling increases productivity and sales: The stable scheduling study. University of California Hastings College of the Law, University of Chicago School of Social Service Administration, University of California Kenan-Flagler Business School.

[R79] Zhang L. (2022). Regulatory spillover and workplace racial inequality. Administrative Science Quarterly, 67(3), 595–629. 10.1177/00018392221085677

