# Supplementary material for: Beyond borders: Does firm-level exposure to state and local paid sick leave mandates lead to intra-firm spillovers?
Source: J Policy Anal Manage. Author manuscript; Available in PMC 2026 Oct 6. (PMC13637550; doi:10.1002/pam.70061)
Supplement: Supplementary file 1 [file NIHMS2209914-supplement-supplementary_material.pdf]

Beyond Borders: Does Firm-Level Exposure to State and Local Paid Sick Leave Mandates Lead to Policy Diffusion?

Online Appendix

Daniel Schneider
Harvard Kennedy School

Kristen Harknett
University of California, San Francisco

Assigning Place of Work Location to Shift Project Respondents

The Shift Project data provide several ways of ascertaining respondents' place of work. First, the survey asks respondents to report on their exact place of work, with fields for store number, store address, store phone number, store cross-streets, and store city/town. In order to match respondents to establishments using this information, we constructed a complete list of every establishment of each of the firms represented in the Shift Project including store number, store address, and store phone number data using store scraping scripts and by purchasing store databases from a vendor (ScrapeHero). We then match respondent reports of establishment information against this list. Of the respondents who provided this information, we were able make an exact match on firm-by-store number for 62% of the sample. We were able to match an additional 6% of the sample using firm-by-store-phone-number or because a firm only had a single location in a city/town. Finally, we match an additional 3% of respondents using string similarity matching (Yancey, 2005; Chetty et al. 2022). In total, we match 71% of respondents to an exact establishment. Second, for the remaining respondents who were surveyed in waves 8-10, we can draw on a question that asked for a self-report of state of work. This give us information for an additional 10% of respondents, though we cannot detect respondents who work in counties with PSL mandates that are in states without PSL mandates using this measure. Finally, for respondents in waves 3-7 who were not matched on establishment as well as for respondents in waves 8-10 who were not matched on establishment and did not provide state of work, we geo-code the IP addresses (collected at the time of survey) of the remaining 19% of respondents and assign county and state of work.

We then define a key variable that captures whether the respondent works in either a county or a state at a time in which a state or county PSL mandate is in effect. We use this measure to

limit our key models to only respondents who are in jurisdictions that do not have a PSL mandate in effect at the time of survey.

Control Variables

Worker-Level Controls. We define a set of worker control variables from survey responses in the Shift data. We control for a set of worker demographics that includes gender, race/ethnicity (white, non-Hispanic; Black, non-Hispanic; Hispanic, and non-Hispanic workers of other or multiple race), age, parental status, speaking language other than English at home, school enrollment, and marital status. We also control for managerial status, job tenure, union coverage, hourly wage, and usual work hours, which may shape access to PSL. We report means of these variables in Appendix Table 6.

Firm-Level Controls. We are somewhat limited in the firm-level attributes that we can control for in the models given that the 135 firms represented in the data are a combination of publicly-held firms, for which there are substantial annual disclosures, as well as privately-held firms, for which only very limited data is available. We control for firm industry (building supplies, general merchandise, grocery stores, auto parts, clothing, furniture, electronics, restaurants, pharmacies, sporting goods, miscellaneous retail) as well as for total firm size in terms of number of employees and number of establishments, calculated from the RefUSA data.

State-Level Controls. We construct a set of time-varying measures of state-level characteristics from various data sources. We control for percent of the population in a union (Current Population Survey), unemployment rate (Current Population Survey), minimum wage (UC Berkeley Labor Center), and demographics by race, age, and gender (American Community Survey).

Figure A1: Share of Workers in Non-PSL Mandate Places Reporting Access to Fringe Benefits, by Firm Exposure to PSL Mandates

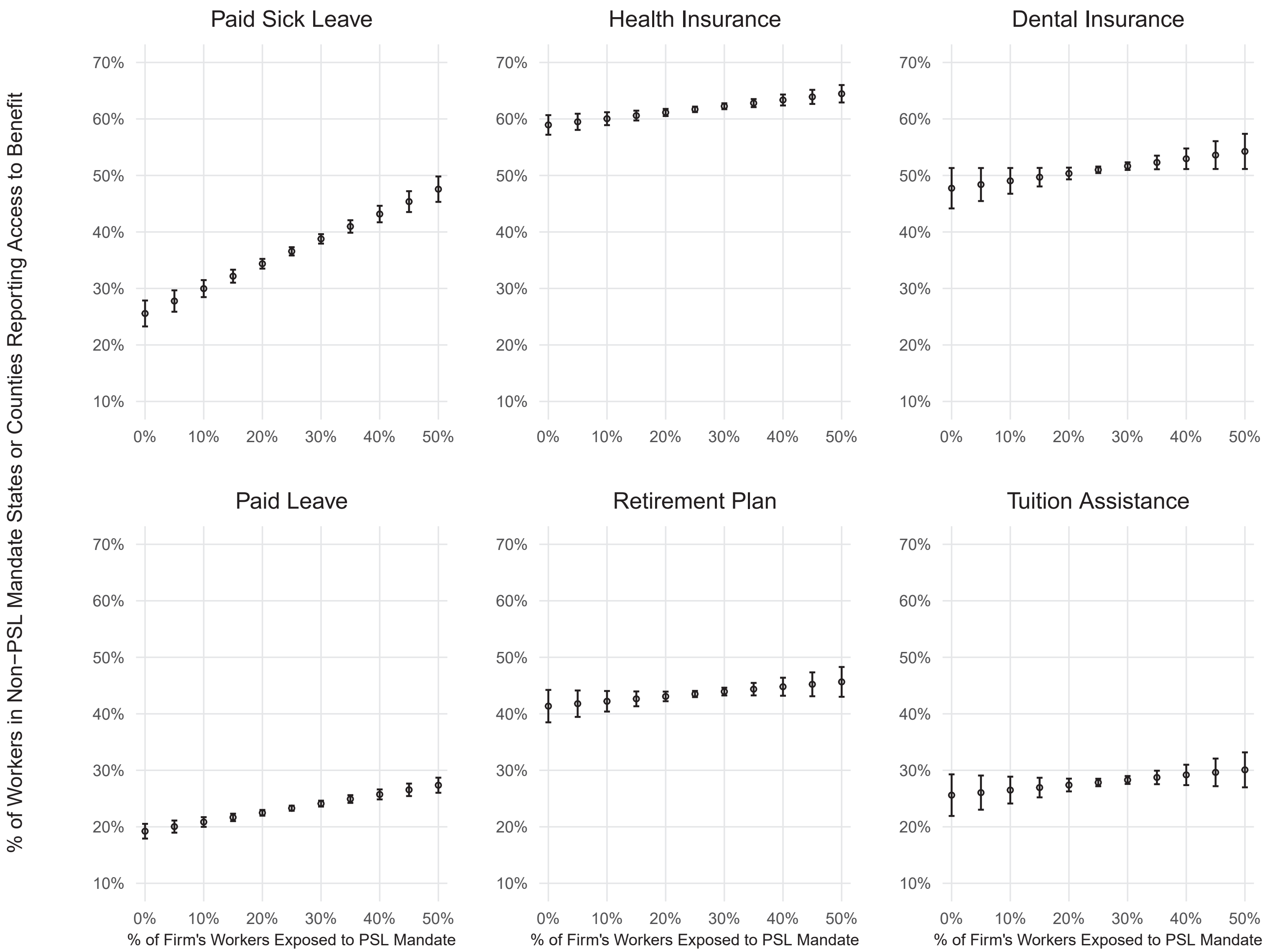

Notes: Predicted values from linear regression model, with the percentage of firm's workers in a PSL mandate place as a continuous regressor. Demographic controls: gender, race, age, parental status, speaking a non-English language at home, being enrolled in school, cohabitation status. Work controls: hourly wage, usual weekly hours, manager status, union membership, job tenure. Also includes state and year/month/year*month fixed effects. Robust standard errors clustered at state level.

Source: Shift Project data, 2018-2021.

Figure A2: Predicted Share of Workers in Non-PSL Mandate Places Reporting Access to Paid Sick by Firm Exposure to PSL Mandates, from Non-Linear Specification

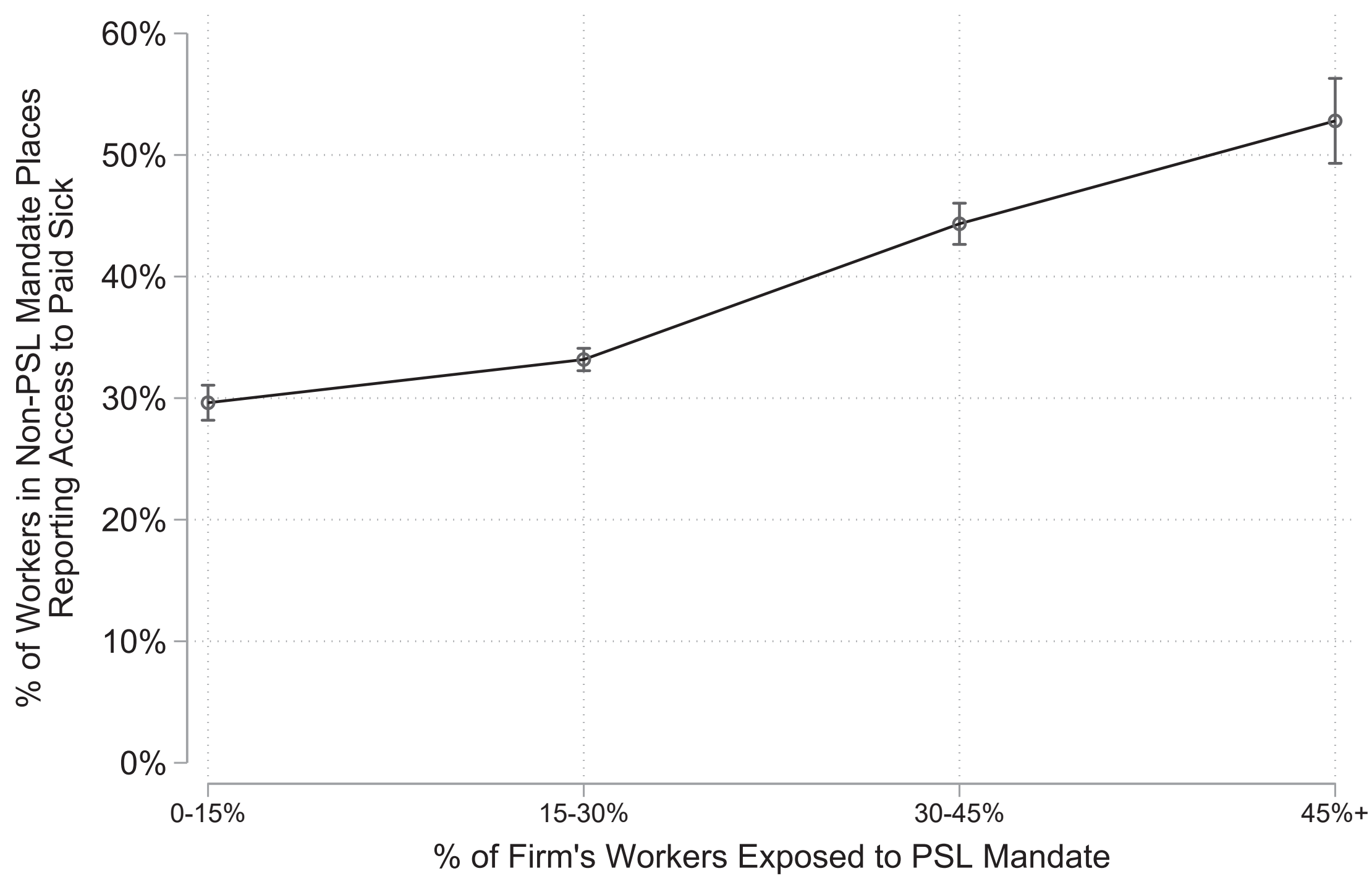

Notes: Predicted values from linear regression model, with the percentage of firm's workers in a PSL mandate place as a categorical regressor. Demographic controls: gender, race, age, parental status, speaking a non-English language at home, being enrolled in school, cohabitation status. Work controls: hourly wage, usual weekly hours, manager status, union membership, job tenure. Also includes state and year/month/year*month fixed effects. Robust standard errors clustered at state level.

Source: Shift Project data, 2018-2021.

Figure A3: Moderation of Association Between Firm-Level Exposure to PSL Mandates and Workers' Reports of Access to PSL in Non-PSL Mandate Places —Firm Headquarters Location

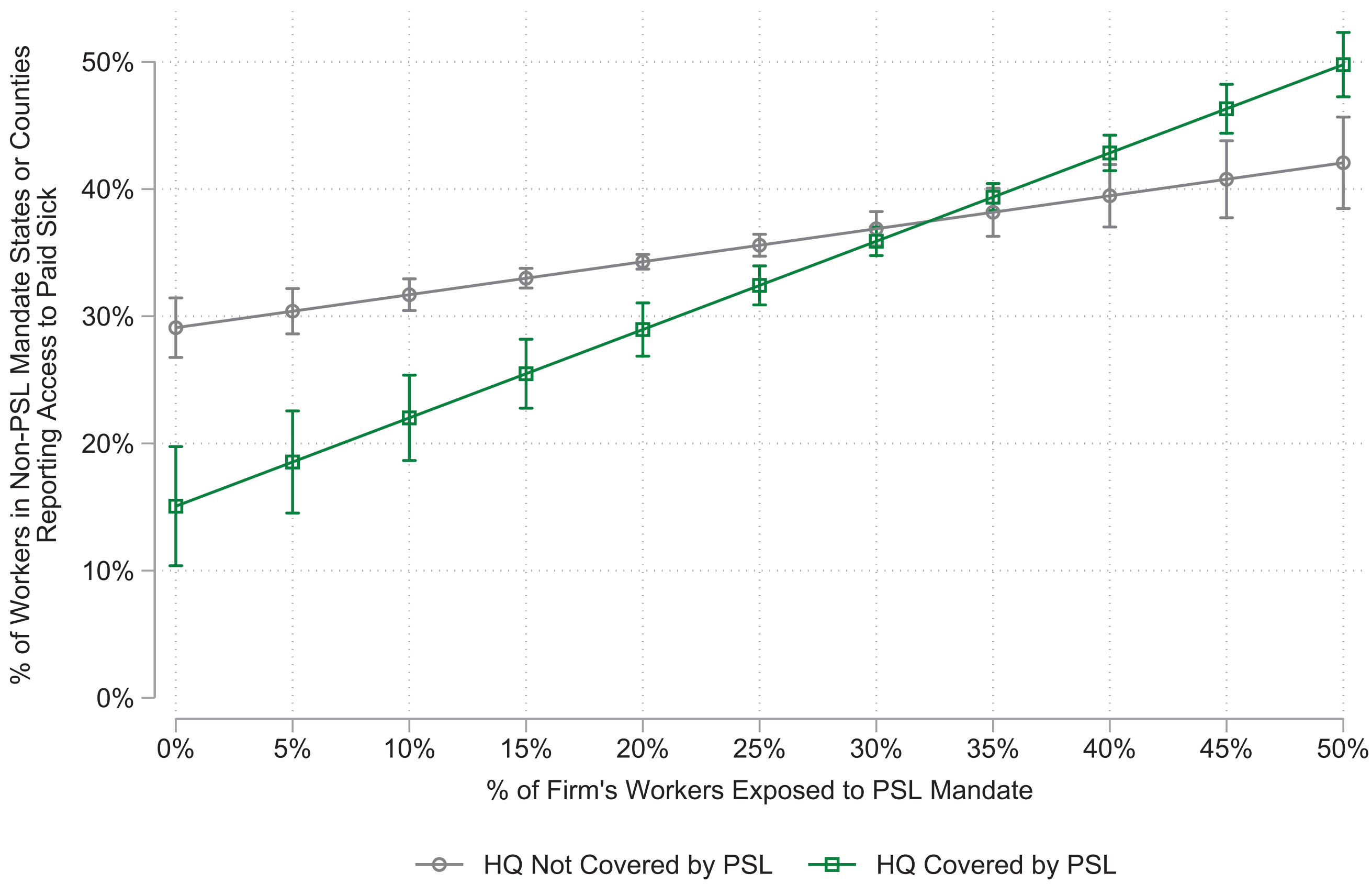

Notes: Predicted values from linear regression model, with the percentage of firm's workers in a PSL mandate place as a continuous regressor in interaction with a binary indicator of whether the firm's headquarters are exposed to a PSL mandate. Demographic controls: gender, race, age, parental status, speaking a non-English language at home, being enrolled in school, cohabitation status. Work controls: hourly wage, usual weekly hours, manager status, union membership, job tenure. Also includes state and year/month/year*month fixed effects. Robust standard errors clustered at state level.

Source: Shift Project data, 2018-2021.

Figure A4: Moderation of Association Between Firm-Level Exposure to PSL Mandates and Workers' Reports of Access to PSL in Non-PSL Mandate Places —Firm Franchising

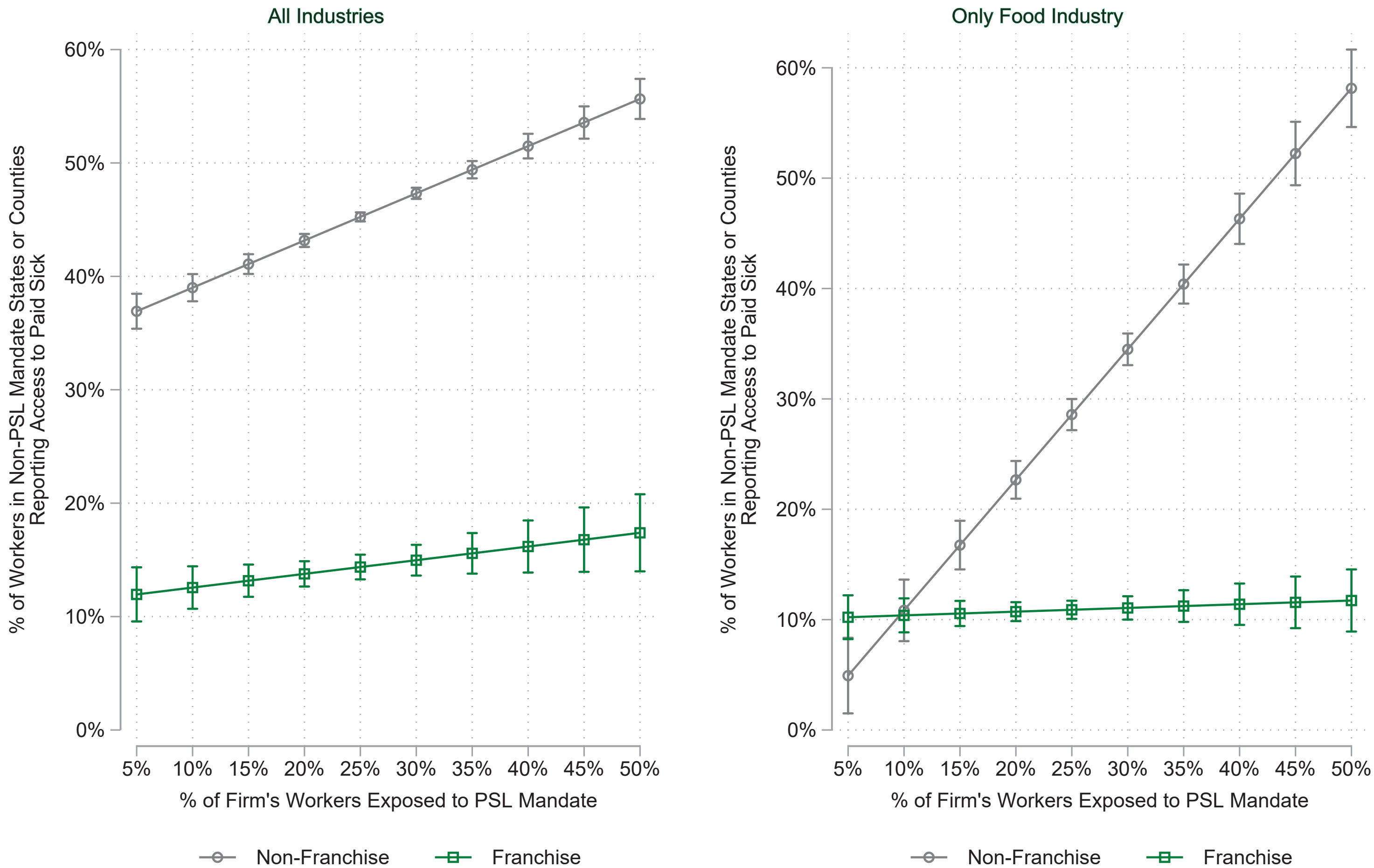

Notes: Predicted values from linear regression model, with the percentage of firm's workers in a PSL mandate place as a continuous regressor in interaction with a binary indicator of whether the firm franchises or not. Demographic controls: gender, race, age, parental status, speaking a non-English language at home, being enrolled in school, cohabitation status. Work controls: hourly wage, usual weekly hours, manager status, union membership, job tenure. Also includes state and year/month/year*month fixed effects. Robust standard errors clustered at state level.

Source: Shift Project data, 2018-2021.

Table A1: Robustness of Association Between Firm-Level Exposure to PSL Mandates and Workers' Reports of Access to PSL in Non-PSL Mandate Places —Excluding 2020/2021 & ACS Weighting

	PSL Access in Non-Mandate Places		
	M1	M2	M3
Share in a PSL Covered State or County			
Percent of Workers	0.005***	0.005***	0.005***
Percent of Establishments	0.005***	0.005***	0.005***
Demographic Controls	✓	✓	✓
Work Controls	✓	✓	✓
Firm Controls	✓	✓	✓
State Controls	✓	✓	✓
State FE	✓	✓	✓
Year, Month, Year × Month FE	✓	✓	✓
**Excluding 2020/2021**		✓	
**ACS Weighting**			✓
N	29382	19576	29381

Notes: M2 excludes observations from 2020 & 2021 from analytic sample. M3 employs American Community Survey weighting on the basis of age, gender, and race. All models include demographic controls, work controls, firm controls, state controls, state fixed effects, and year/month/year*month fixed effects. Demographic controls: gender, race, age, parental status, speaking a non-English language at home, being enrolled in school, cohabitation status. Work controls: hourly wage, usual weekly hours, manager status, union membership, job tenure. Firm controls: industry, total number of employees (by month), total number of establishments (by month). State controls: unemployment rate, minimum wage, union membership, breakdowns of age/gender/race/education. Robust standard errors clustered at state level.

Source: Shift Project data, 2018-2021.

Table A2: Association Between Firm-Level Exposure to PSL Mandates and Workers' Reports of Access to PSL in Non-PSL Mandate Places

	Panel A: Workers in non-PSL States						Panel B: Workers in non-PSL States or Counties					
	M1	M2	M3	M4	M5	M6	M7	M8	M9	M10	M11	M12
Share in a PSL Covered State												
Percent of Workers	0.006***	0.004***	0.005***	0.005***	0.005***	0.005***	0.006***	0.004***	0.005***	0.005***	0.005***	0.005***
Percent of Establishments	0.006***	0.004***	0.005***	0.005***	0.005***	0.005***	0.006***	0.004***	0.005***	0.005***	0.005***	0.005***
Share in a PSL Covered State or County												
Percent of Workers	0.006***	0.004***	0.005***	0.005***	0.005***	0.005***	0.006***	0.004***	0.005***	0.005***	0.005***	0.005***
Percent of Establishments	0.006***	0.004***	0.005***	0.005***	0.005***	0.005***	0.006***	0.004***	0.005***	0.005***	0.005***	0.005***
Demographic Controls	✓	✓	✓	✓	✓	✓	✓	✓	✓	✓	✓	✓
Work Controls		✓	✓	✓	✓	✓		✓	✓	✓	✓	✓
State FE			✓	✓	✓	✓			✓	✓	✓	✓
Year, Month, Year × Month FE				✓	✓	✓				✓	✓	✓
State Controls					✓	✓					✓	✓
Firm Controls						✓						✓
N	31459	31459	31459	31459	31459	31459	29382	29382	29382	29382	29382	29382

$^{+}$ $p < 0.10$, * $p < 0.05$, ** $p < 0.01$, *** $p < 0.001$

Notes: Demographic controls: gender, race, age, parental status, speaking a non-English language at home, being enrolled in school, cohabitation status. Work controls: hourly wage, usual weekly hours, manager status, union membership, job tenure. Firm controls: industry, total number of employees (by month), total number of establishments (by month). State controls: unemployment rate, minimum wage, union membership, breakdowns of age/gender/race/education. Robust standard errors clustered at state level.

Source: Shift Project data, 2018-2021.

Table A3: Robustness of Association Between Firm-Level Exposure to PSL Mandates and Workers' Reports of Access to PSL in Non-Mandate Places to Alternative Treatment of PTO States

	PSL Access in Non-Mandate States			PSL Access in Non-Mandate States/Counties		
	M1	M2	M3	M4	M5	M6
Share in PSL Covered State						
Percent of Workers	0.005***			0.005***		
Percent of Establishments	0.005***			0.005***		
Share in PSL Covered State or County						
Percent of Workers	0.005***			0.005***		
Percent of Establishments	0.005***			0.005***		
Share in PSL Covered State						
Percent of Workers		0.005***			0.004***	
Percent of Establishments		0.005***			0.005***	
Share in PSL Covered State or County						
Percent of Workers		0.005***			0.004***	
Percent of Establishments		0.005***			0.005***	
Share in PSL Covered State						
Percent of Workers			0.005***			0.005***
Percent of Establishments			0.005***			0.005***
Share in PSL Covered State or County						
Percent of Workers			0.005***			0.005***
Percent of Establishments			0.005***			0.005***
Maine/Michigan/Nevada = no PSL	✓			✓		
Maine/Michigan/Nevada = PSL		✓			✓	
Maine/Michigan/Nevada = dropped			✓			✓
N	31459	30517	30517	29382	28453	28453

Notes: M1 & M4 treat states with PTO mandates (ME/MI/NV) as PSL mandate states. M2 & M5 treat states with PTO mandates (ME/MI/NV) as non-PSL mandate states. M3 & M6 omit PTO states (ME/MI/NV) from model. All models include demographic controls, work controls, firm controls, state controls, state fixed effects, and year/month/year*month fixed effects. Demographic controls: gender, race, age, parental status, speaking a non-English language at home, being enrolled in school, cohabitation status. Work controls: hourly wage, usual weekly hours, manager status, union membership, job tenure. Firm controls: industry, total number of employees (by month), total number of establishments (by month). State controls: unemployment rate, minimum wage, union membership, breakdowns of age/gender/race/education. Robust standard errors clustered at state level.

Source: Shift Project data, 2018-2021.

Table A4: Share of Workers in Non-PSL Mandate Places Reporting Access to Fringe Benefits, by Firm Exposure to Mandates

	PSL Access in Non-Mandate Places					
	Paid Sick Leave	Health Insurance	Dental Insurance	Paid Leave	Retirement Plan	Tuition Assistance
Share in a PSL Covered State or County						
Percent of Workers	0.005***	0.002***	0.002***	0.002***	0.002***	0.001*
Demographic Controls	✓	✓	✓	✓	✓	✓
Work Controls	✓	✓	✓	✓	✓	✓
Firm Controls	✓	✓	✓	✓	✓	✓
State Controls	✓	✓	✓	✓	✓	✓
State FE	✓	✓	✓	✓	✓	✓
Year, Month, Year × Month FE	✓	✓	✓	✓	✓	✓
N	29382	29382	29382	29382	29382	29382

$^{+}$ $p < 0.10$, * $p < 0.05$, ** $p < 0.01$, *** $p < 0.001$

Notes: All models include demographic controls, work controls, firm controls, state controls, state fixed effects, and year/month/year*month fixed effects. Demographic controls: gender, race, age, parental status, speaking a non-English language at home, being enrolled in school, cohabitation status. Work controls: hourly wage, usual weekly hours, manager status, union membership, job tenure. Firm controls: industry, total number of employees (by month), total number of establishments (by month). State controls: unemployment rate, minimum wage, union membership, breakdowns of age/gender/race/education. Robust standard errors clustered at state level.

Source: Shift Project data, 2018-2021.

Table A5: Association Between Firm-Level Exposure to PSL Mandates and Workers' Reports of Access to PSL in Non-PSL Mandate Places, from Non-Linear Specification

	PSL Access in Non-Mandate Places					
	M1	M2	M3	M4	M5	M6
Share in a PSL Covered State or County						
15-30% of Workers Covered	0.024^{+}	0.021^{*}	0.022^{*}	0.032^{**}	0.032^{**}	0.033^{**}
30-45% of Workers Covered	0.170^{***}	0.122^{***}	0.116^{***}	0.127^{***}	0.127^{***}	0.130^{***}
45%+ of Workers Covered	0.284^{***}	0.206^{***}	0.263^{***}	0.278^{***}	0.277^{***}	0.275^{***}
Demographic Controls	✓	✓	✓	✓	✓	✓
Work Controls		✓	✓	✓	✓	✓
Firm Controls			✓	✓	✓	✓
State Controls				✓	✓	✓
State FE					✓	✓
Year, Month, Year × Month FE						✓
N	29382	29382	29382	29382	29382	29382

$^{+}$ $p < 0.10$, * $p < 0.05$, ** $p < 0.01$, *** $p < 0.001$

Notes: Reference category for share of workers covered by PSL mandate: 0-15%. Demographic controls: gender, race, age, parental status, speaking a non-English language at home, being enrolled in school, cohabitation status. Work controls: hourly wage, usual weekly hours, manager status, union membership, job tenure. Firm controls: industry, total number of employees (by month), total number of establishments (by month). State controls: unemployment rate, minimum wage, union membership, breakdowns of age/gender/race/education. Robust standard errors clustered at state level.

Source: Shift Project data, 2018-2021.

Table A6: Descriptive Statistics

	Mean / %
Demographic Characteristics	
Age	36.6
Gender	
Male	27
Female	73
Race/Ethnicity	
White	83
Black	3
Hispanic	8
Other / 2+ Races	6
Cohabitation Status	
Married, Living with Spouse	29
Living with Partner	20
Not Living with Spouse/Partner	51
Has Children	45
Speaks Non-English Language at Home	9
Enrolled in School	24
Work Characteristics	
Hourly Wage	$12.08
Usual Weekly Work Hours	32.4
Job Tenure	
1 Year or Less	34
2-3 Years	26
4-5 Years	13
6+ Years	28
Manager	24
Union Member	6
Industry	
Building Supplies / Hardware	6
General Merchandise	19
Grocery Stores	17
Auto Parts	2
Clothing	3
Home Furnishings	1
Electronics Stores	2
Restaurants / Food Service	38
Pharmacies / Drug Stores	5
Sporting Goods / Toy Stores	2
Misc. Retail	5
N	29382

Notes: Race/ethnicity categories are mutually exclusive; i.e., respondents selecting Hispanic in addition to other races/ethnicities are classified as Hispanic.

Source: Shift Project data, 2018-2021.

Table A7: Moderation of Association between Firm-Level Exposure to PSL Mandates and Workers' Reports of Access to PSL in Non-Mandate Places —Firm Headquarters Location, Excluding IKEA

	PSL Access in Non-Mandate Places
Share in PSL Covered State or County	
Percent of Workers	0.002**
Firm Headquartered in PSL Mandated Place	-0.053+
Percent of Workers Covered by PSL × HQ Covered	0.004***
Share in PSL Covered State or County	
Percent of Establishments	0.002**
Firm Headquartered in PSL Mandated Place	-0.064+
Percent of Establishments Covered by PSL × HQ Covered	0.005***
Observations	29317

+ $p < 0.10$, * $p < 0.05$, ** $p < 0.01$, *** $p < 0.001$

Notes: Dependent variable: reported access to PSL among workers in states/counties not covered by PSL mandates. Excludes all observations from workers at IKEA. All models include demographic controls, work controls, firm controls, state controls, state fixed effects, and year/month/year*month fixed effects. Demographic controls: gender, race, age, parental status, speaking a non-English language at home, being enrolled in school, cohabitation status. Work controls: hourly wage, usual weekly hours, manager status, union membership, job tenure. Firm controls: industry, total number of employees (by month), total number of establishments (by month). State controls: unemployment rate, minimum wage, union membership, breakdowns of age/gender/race/education. Robust standard errors clustered at state level.

Source: Shift Project data, 2018-2021.

Figure A5: Moderation of Association Between Firm-Level Exposure to PSL Mandates and Workers' Reports of Access to PSL in Non-PSL Mandate Places —Firm Headquarters Location, Excluding IKEA

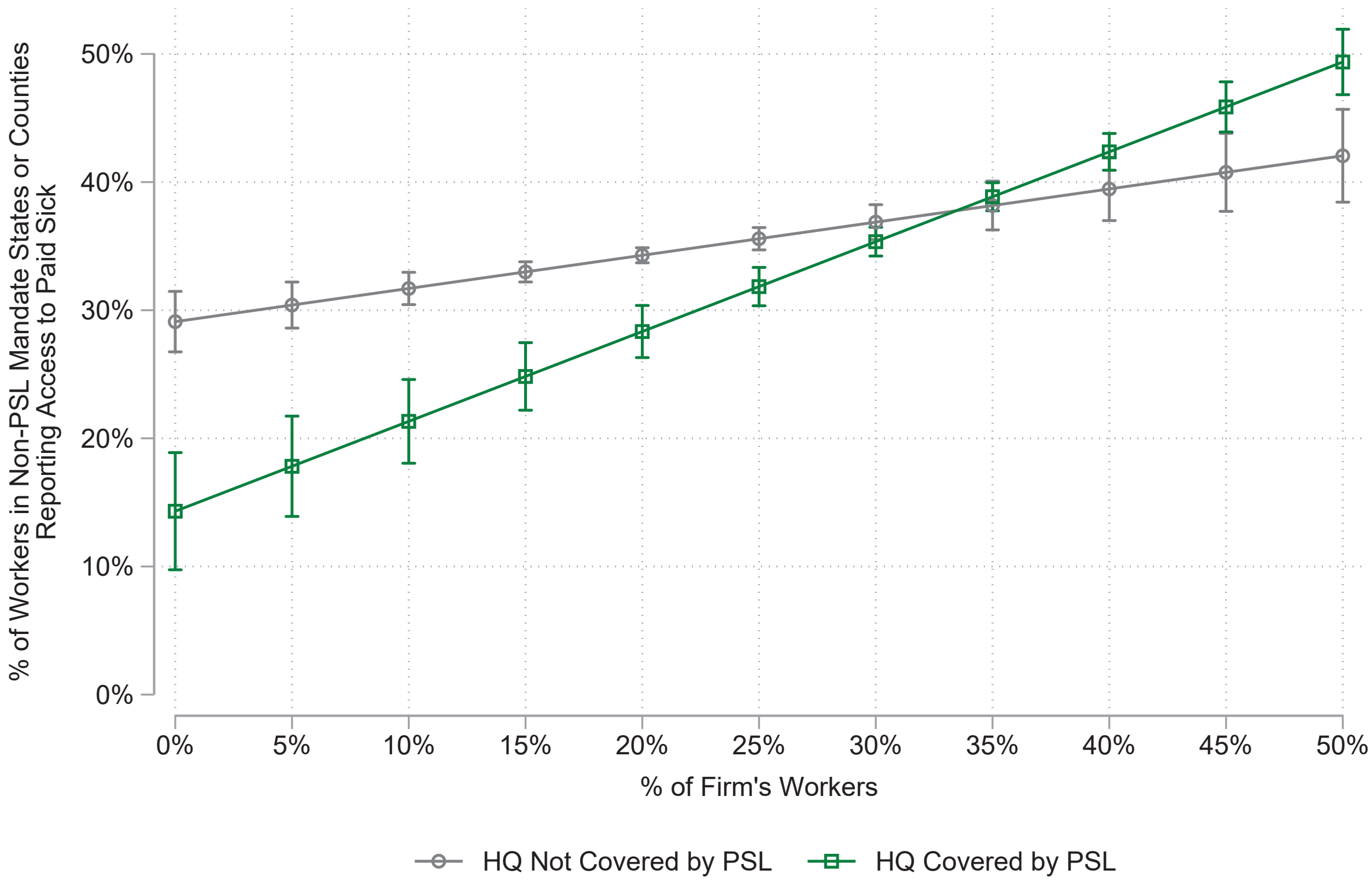

Notes: Predicted values from linear regression model, with the percentage of firm's workers in a PSL mandate place as a continuous regressor in interaction with a binary indicator of whether the firm's headquarters are exposed to a PSL mandate. Excludes all observations from workers at IKEA. Demographic controls: gender, race, age, parental status, speaking a non-English language at home, being enrolled in school, cohabitation status. Work controls: hourly wage, usual weekly hours, manager status, union membership, job tenure. Also includes state and year/month/year*month fixed effects. Robust standard errors clustered at state level.

Source: Shift Project data, 2018-2021.
